# Laeverin is Cell‐Surface Target for Liquid‐Phase Metastasizing Cancer Cells

**DOI:** 10.1002/advs.202511349

**Published:** 2025-09-29

**Authors:** Haruki Kasama, Yuya Sakai, Kyosuke Kagami, Takashi Iizuka, Tatsuhito Kanda, Takuma Suzuki, Kayo Kayahashi, Masanori Ono, Tomoko Fujiwara, Shintaro Yagi, Noriyuki Inaki, Isao Matsumoto, Rena Yamazaki, Kaoru Abiko, Noriomi Matsumura, Akira Hattori, Takiko Daikoku, Hiroshi Fujiwara

**Affiliations:** ^1^ Department of Obstetrics and Gynecology Graduate School of Medical Sciences Kanazawa University Kanazawa Ishikawa 920‐8641 Japan; ^2^ Department of Obstetrics and Gynecology Tokyo Medical University Nishi‐Shinjuku Shinjuku‐ku Tokyo 160‐0023 Japan; ^3^ Department of Human Life Environments Kyoto Notre Dame University Kyoto 606‑0847 Japan; ^4^ Department of Hepato‐Biliary‐Pancreatic Surgery and Transplantation Kanazawa University Hospital Kanazawa 920‑8641 Japan; ^5^ Department of Gastrointestinal Surgery Kanazawa University Kanazawa 920‐8641 Japan; ^6^ Department of Thoracic Surgery Kanazawa University Hospital Kanazawa 920‐8641 Japan; ^7^ Department of Obstetrics and Gynecology Kindai University Faculty of Medicine Osaka 589‑8511 Japan; ^8^ Department of System Chemotherapy and Molecular Sciences Kyoto University Graduate School of Pharmaceutical Sciences Kyoto 606‐8501 Japan; ^9^ Division of Animal Disease Model Research Center for Experimental Modeling of Human Disease Kanazawa University Kanazawa Ishikawa 920‐8640 Japan; ^10^ Ochi Yume Clinic Nagoya Nagoya 460‐0002 Japan; ^11^ School of Veterinary Medicine Azabu University Sagamihara 252‐5201 Japan

**Keywords:** antigen‐drug conjugate, cancer stem cell, distant metastasis, laeverin, liquid phase, Oct4, spheroid

## Abstract

Laeverin (LVRN) is a cell‐surface immunoregulatory factor that is specifically expressed in embryo‐derived extravillous trophoblast, which invades maternal spiral arteries without immune rejection during human placentation. Here, it is found that various epithelial cancer cell lines upregulated LVRN expression in association with the expression of *POU5F1* after spheroid formation under floating conditions. Immunohistochemically, LVRN expression is detected in the lesions of vascular, lymphatic, and peritoneal invasion of ovarian, cervical, endometrial, breast, and lung cancers. LVRN‐positive circulating tumor cells are also identified in the blood of uterine cervical and endometrial cancers, showing that LVRN expression is induced in cancer cells in the distant metastatic phase. Monomethylauristatin E‐conjugated anti‐LVRN antibody induced cell death in ovarian cancer‐derived cell lines in the liquid phase in vitro and inhibited their peritoneal dissemination in nude mice in vivo. These findings indicate that LVRN is a unique and promising cell‐surface target molecule for liquid‐phase metastasizing cancer cells.

## Graphical abstract

This study revealed that epithelial cancer cells in the floating phase induce the expression of laeverin (LVRN) that is a trophoblast‐specific and cell‐surface immunoregulatory factor and showed that anti‐LVRN ADC is a promising agent that can attack cancer cells in the metastatic liquid phase and block further distant metastasis.

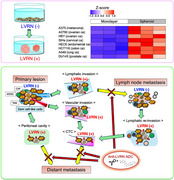

## 1 Introduction

Two decades ago, we identified laeverin/aminopeptidase Q(AQPEP) from human placenta.^[^ ^1^ ^]^ LVRN is a type II membrane‐bound peptidase that belongs to the M1peptidase family.^[^ ^2^ ^]^ In contrast to other aminopeptidases, LVRN expression is restricted to the extravillous trophoblast that invades the maternal vessels during human placentation. Although the extravillous trophoblast has allogeneic paternal antigens, it acquires immunotolerance and is not rejected by the maternal immune system, forming the layer of maternal‐fetal interface throughout the uterus.^[^ ^3^ ^]^

No apparent expression of LVRN was detected in normal organs other than placenta, including various malignant or non‐malignant cell lines.^[^ ^1^ ^]^ The primate LVRN has a novel peptide binding site.^[^ ^2^ ^]^ Unlike humans, murine LVRN is widely expressed in various organs,^[^ ^4^ ^]^ suggesting that human LVRN has acquired distinct functions from those of other animal species during evolution. Later, we found that LVRN directly interacts with monocytes to produce an immunosuppressive molecule, indoleamine 2,3‐dioxygenase‐1 (IDO1), and elevates the extracellular concentration ratio of kynurenine/tryptophan around monocytes, indicating that LVRN is a novel embryonic signal that can create an immunosuppressive environment at the feto‐maternal interface.^[^ ^5^ ^]^

Extravillous trophoblast is considered to differentiate from a cytotrophoblast, and we previously observed that LVRN expression was induced during this differentiation process in vitro.^[^ ^6^ ^]^ Recently, we found that Swan71 cells, a human cytotrophoblast‐derived cell line, promoted LVRN expression during spheroid formation under floating culture conditions.^[^ ^7^ ^]^ In general, epithelial cancer cells need to transition to a floating condition and move through liquid environments to achieve distant metastasis. Therefore, in this study, we examined the induction of LVRN expression in spheroid‐formed epithelial cancer cell lines and observed LVRN expression in clinical samples of various epithelial cancer cells undergoing different clinical phases or conditions. Furthermore, we investigated the therapeutic potential of an antigen‐drug conjugate (ADC) targeting LVRN in mouse models.

## 2 Results

### 2.1 LVRN Expression was Induced in Various Epithelial Cancer Cell Lines

**Figure 1:**
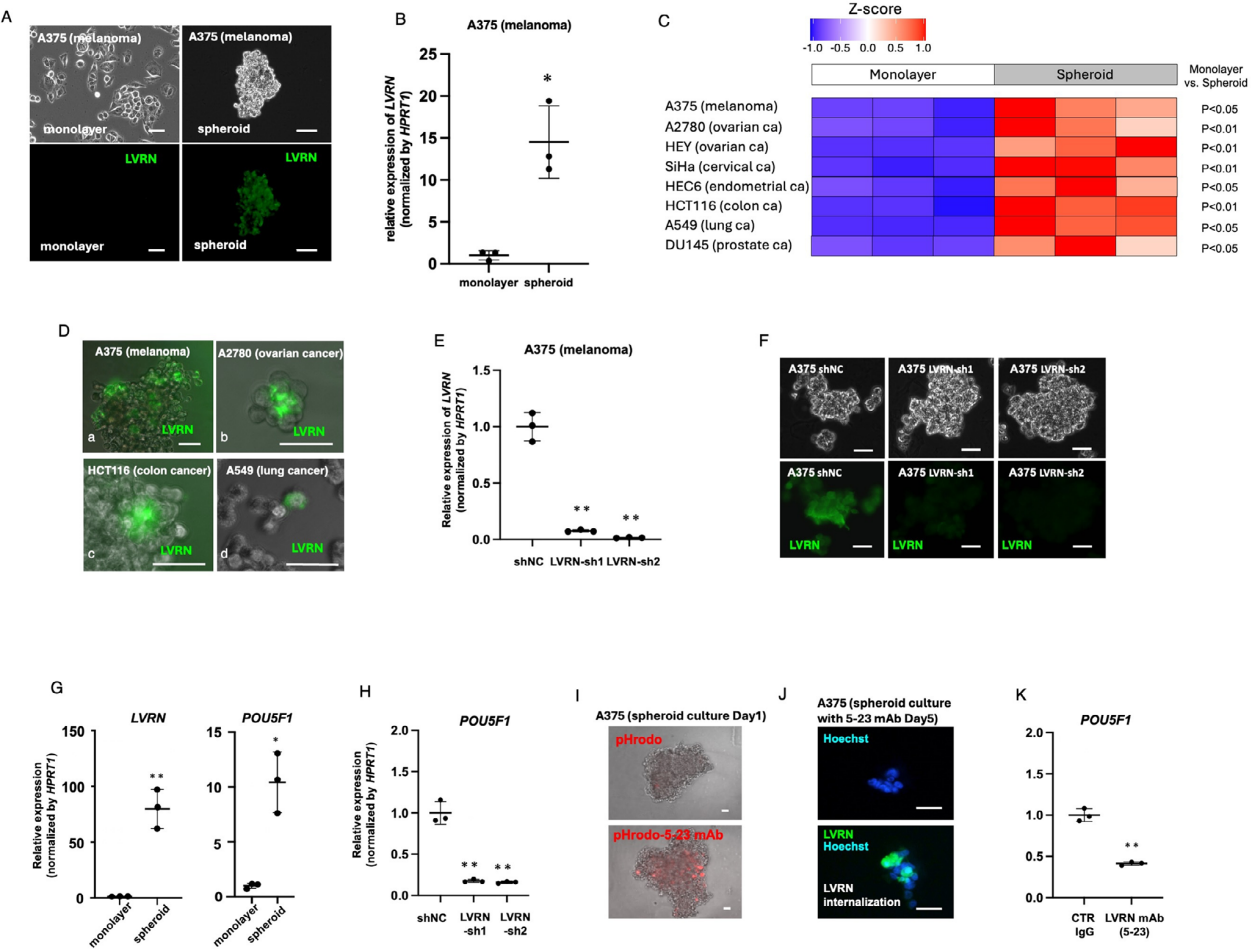
Expression of LVRN and its associated genes in cancer cell lines. A,B) Monolayer and spheroid‐cultured A375 cells. Immunofluorescent (A) and mRNA (B) expression of LVRN was induced by spheroid culture. C) mRNA expression of LVRN was induced by spheroid culture in various cancer cell lines. D) LVRN was also induced by hanging culture. E) LVRN expression in A375 cells was knocked‐down by LVRN‐sh1 and LVRN‐sh2. F) LVRN induction was lost in spheroid‐cultured knocked‐down A375 cells. Spheroid culture increased mRNA expressions of LVRN and *POU5F1* in wild‐type A375 cells (G), whereas this increase was attenuated in spheroid‐formed A375‐LVRN‐KD cells (H). I–K) pHrodo‐conjugated anti‐LVRN mAb was internalized into spheroid cultured wild‐type A375 cells (I). This spheroid‐forming culture with 5–23 mAb also induced internalization of LVRN (J) and reduced *POU5F1* expression (K). Bars show 50 µm. Statistical analysis was performed by Student's t‐test and One‐way ANOVA with post‐hoc Tukey's multiple comparisons test. *p<0.05, **p<0.01. Error bars represent standard deviation.

Since melanoma tends to undergo vascular metastasis, we first investigated a melanoma‐derived cell line, A375. Immunocytochemical staining using a mouse anti‐human LVRN mAb (clone 5–23) raised in our laboratory^[^ ^1^ ^]^ showed that A375 cells were negative for LVRN under monolayer‐culture, whereas they came to express LVRN on their cell surfaces after 3‐day spheroid‐forming culture (**Figure** **1**A). By qPCR, the mRNA expression of LVRN also increased by the spheroid formation (Figure 1B). Similar expression profiles were observed in various epithelial cancer cell lines, such as ovarian, cervical, endometrial, breast, colorectal, lung, and prostate cancers (Figure 1C; Figure S1A–D, Supporting Information). The increase of LVRN expression in spheroid‐forming cancer cell lines was confirmed by other mouse anti‐human LVRN mAbs (clones 5–23J, 92–4, and 129‐5, raised in our laboratory) (Figure S1E, Supporting Information). After 1‐day hanging drop culture,^[^ ^8^ ^]^ LVRN expression was also induced in floating cancer cell lines (Figure 1D).

### 2.2 LVRN Expression was Associated with Stem Cell and Cell‐Cycle‐Related Gene Expressions

To examine the LVRN‐associated genes, we knocked down LVRN in A375 cells by shRNAs (A375‐LVRN‐KD cells) (Figure 1E,F) and compared gene expression profiles with those of the wild‐type A375 cells under spheroid formation. As a stem cell‐related transcription factor gene, qPCR showed increase in *POU5F1* (*OCT4*) expression in spheroid‐formed wild‐type A375 cells (Figure 1G) and revealed that this increase was attenuated in A375‐LVRN‐KD cells (Figure 1H). Using a pH sensor probe (pHrodo Red) that indicates phagocytosis, pHrodo‐conjugated anti‐LVRN mAb (5‐23) was demonstrated to be internalized into spheroid cultured wild‐type A375 cells (Figure 1I). This spheroid‐forming culture with 5–23 mAb also induced internalization of LVRN (Figure 1J) and reduced *POU5F1* expression (Figure 1K), confirming the involvement of LVRN in *POU5F1* induction.

In human iPS cells, mRNA expression of LVRN was detected (Figure S2A–C, Supporting Information). When iPS cells were cultured under conditions to induce neural differentiation, LVRN expression decreased during the process of differentiation (Figure S2D, Supporting Information), suggesting a correlation between LVRN and stem cell properties.

In HCT116 cells (colorectal cancer) and A549 cells (lung cancer), whose *LVRN* was knocked out by the CRISPR‐Cas9 system, *POU5F1* expression was also shown to be associated with that of LVRN (Figure S3A–F, Supporting Information). In two ovarian cancer cell lines A2780 and HEY cells, reductions of *LVRN* expression by shRNAs were associated with decreases in *POU5F1* expression (Figure S3G–L, Supporting Information).

### 2.3 LVRN was Expressed in the Liquid Phase of Cancer Cells in Clinical Samples

**Figure 2:**
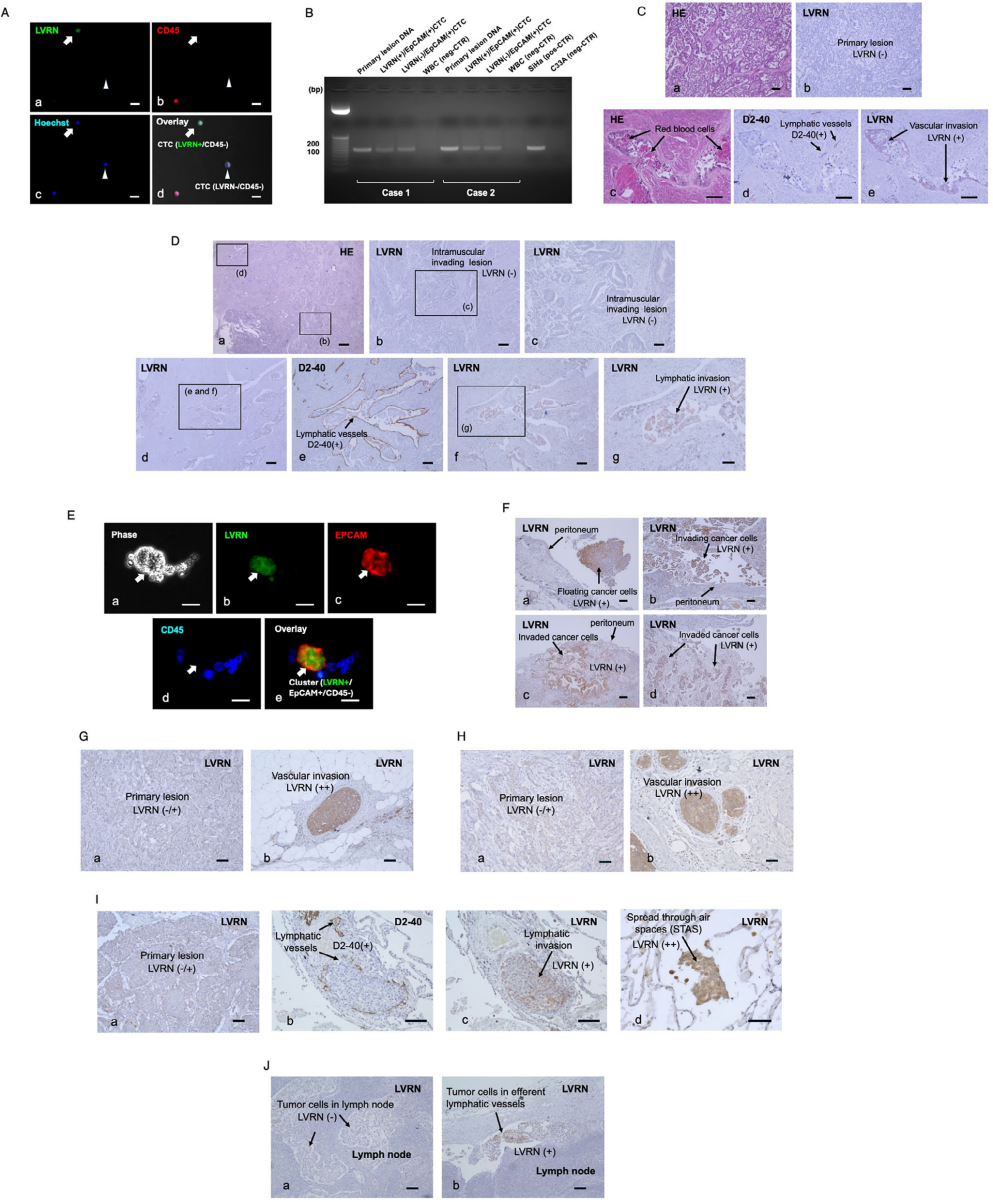
LVRN expression in gynecological cancers. A–C) Uterine cervical cancers. A) Among circulating cells enriched by anti‐EpCAM Ab, both LVRN‐positive (arrows) and LVRN‐negative CTCs (arrowheads) were detected. B) The integration of the type 16 HPV E6/E7 gene was detected in the whole genomes amplified from EpCAM/LVRN double‐positive cells. C) Positive expression of LVRN was observed in vascular‐invading lesions (c–e), but not in the primary lesion (a,b). D) Uterine endometrial cancer. LVRN expression was not observed in the primary and muscular‐invading lesions (a–c) but was induced in cancer cells (f,g) that invaded D2‐40‐positive lymphatic vessels (e). E,F) Ovarian cancers. E) EpCAM/LVRN double‐positive cancer cells were detected in ascites. F) In other 4 cases, LVRN‐positive cancer cells were observed in the peritoneal‐invading lesions. G) Breast cancer, invasive ductal carcinoma, stage IIB. Primary lesion was very weakly positive (a), whereas vascular‐invading lesions were markedly positive for LVRN (b). H) Breast cancer, invasive ductal carcinoma, stage IIA. Primary lesion was weakly positive (a), whereas vascular‐invading lesions were markedly positive for LVRN (b). I. Lung cancer, large cell carcinoma, stage IIB. Primary lesion was weakly positive for LVRN (a), whereas its expression increased in the lymphatic‐invading lesions ((b,c) thin arrows). Tumor cells spreading through air spaces were markedly positive for LVRN (d). J) Lung cancer, poorly differentiated non‐small cell carcinoma, stage IIIA. Tumor cells in the lymph node were negative for LVRN ((a) thin arrows), whereas positive tumor cells were observed in efferent lymph vessels ((b) thick arrows) in the metastatic lymph node. Bars show D(a)) 1 cm, D(b),D(d)) 200 µm, C,D(c),D(e),D(f),F–J) 100 µm, D(g)) 50 µm, and A,E) 20 µm.

In blood samples from 7 uterine cervical cancer patients (**Table** **1**), LVRN‐expressing cells were identified among circulating tumor cells (CTCs) that were enriched by anti‐epithelial cell adhesion molecule (EpCAM) Ab (**Figure** **2**A). In two cases, we examined integration of the type 16 HPV E6/E7 gene and detected it in the whole genomes amplified from EpCAM/LVRN double‐positive cells, confirming that these LVRN‐positive cells were derived from the HPV‐infected primary tumor (Figure 2B). In another cervical cancer patient (clear cell carcinoma), immunohistochemical expression of LVRN was negative in the primary lesions (Figure 2C(a),(b)), whereas positive cancer cells were observed in vascular‐invading lesions (Figure 2C(c)–(e)).

**Table 1:** Clinical data of patients with various cancers evaluated for LVRN expression by immunocytochemical and immunohistochemical studies.

Case	Age	Organ	Histrology	Stage (TNM)	Primary lesion	Vessel lesion	CTC positivity	Ascitic cells	Peritone‐al lesion	Figures
1	55	Uterine cervix	Adenocarinoma, gastric type	IIIC1p (pT2b pN1 M0)	**–**	**+**	N/E	N/A	N/A	
2	26	Uterine cervix	SCC	IIIC1p (pT2b pN1 M0)	**–**	**+**	N/E	N/A	N/A	
3	43	Uterine cervix	Adenosquamous carcinoma	IIIC1p (pT2b pN1 M0)	**–**	**+**	N/E	N/A	N/A	
4	72	Uterine cervix	Clear cell carcinoma	IIIC1p (pT1b pN1 M0)	**–**	**+**	N/E	N/A	N/A	Figure 2C
5	51	Uterine cervix	SCC	IIIC1p (pT2b pN1 M0)	**–**	**+**	N/E	N/A	N/A	
6	46	Uterine cervix	SCC	IIB (pT2b pN0 M0)	**–**	**+**	N/E	N/A	N/A	
7	73	Uterine cervix	SCC (HPV16+)	IIIC2r (T2b N2 M0)	**–**	N/A	**+** (7/8) 87%	N/A	N/A	
8	57	Uterine cervix	SCC (HPV16+)	IVB (T3b N2 M1)	**−/+**	N/A	**+** (3/56) 5%	N/A	N/A	Figure2A,B case1
9	58	Uterine cervix	SCC (HPV16+)	IVB (T4a N2 M1)	**–**	N/A	**+** (12/23) 52%	N/A	N/A	
10	57	Uterine cervix	SCC (HPV33+)	IVB (T4a N2 M1)	**–**	N/A	**+** (9/19) 47%	N/A	N/A	
11	58	Uterine cervix	SCC (HPV16+)	IVB (T2b N2 M1)	**−/+**	N/A	**+** (6/11) 55%	N/A	N/A	Figure 2B case2
12	71	Uterine cervix	SCC	IVA (T4a N0 M0)	**–**	N/A	**+** (11/42) 26%	N/A	N/A	
13	73	Uterine cervix	Adenocarinoma (HPV‐)	IVB (T2b N2 M1)	**+**	N/A	**+** (9/12) 75%	N/A	N/A	
14	86	Uterine corpus	Endomtetrioid carcinoma, G2	IIIC2 (pT3a pN2 M0)	**–**	**+**	N/E	N/A	N/A	Figure 2D
15	80	Uterine corpus	Endomtetrioid carcinoma, G1	IIIC1 (pT1b pN1 M0)	**–**	**+**	N/E	N/A	N/A	
16	65	Uterine corpus	Endomtetrioid carcinoma, G1	IB (pT1b pN0 M0)	**–**	**+**	N/E	N/A	N/A	Figure S4A (Supporting Information)
17	62	Uterine corpus	Endomtetrioid carcinoma, G2	IIIC2 (pT1b pN2 M0)	**–**	**+**	N/E	N/A	N/A	Figure S4B (Supporting Information)
18	59	Uterine corpus	Endomtetrioid carcinoma, G1	IIIC2 (pT3a pN2 M0)	**–**	**+**	N/E	N/A	N/A	Figure S4C (Supporting Information)
19	74	Uterine corpus	Serous carcinoma	IB (pT1b pN0 M0)	**–**	**+**	N/E	N/A	N/A	
20	65	Uterine corpus	Carcinosarcoma	IB (pT1b pN0 M0)	**–**	**+**	N/E	N/A	N/A	
21	74	Uterine corpus	Endomtetrioid carcinoma, G2	IIIC1 (pT1b pN1 M0)	**−/+**	**+**	**+** (11/15) 73%	N/A	N/A	Figure S4D (Supporting Information)
22	68	Uterine corpus	Carcinosarcoma	IVB (pT3a pN2 M1)	**–**	**−/+**	**+** (5/13) 38%	**+**	N/E	Figure S4E (Supporting Information)
23	58	Ovary	High‐grade serous carcinoma	IVB (T3c N1 M1b)	**–**	N/A	N/E	**+**	N/E	Figure 2E
24	53	Ovary	Clear cell carcinoma	IIIB (pT3b pN0 M0)	**–**	N/A	N/E	**+**	N/E	
25	70	Ovary	High‐grade serous carcinoma	IVA (pT3c N1 M1a)	**–**	N/A	N/E	N/E	**+**	Figure 2F‐a
26	40	Ovary	High‐grade serous carcinoma	IIIC (pT3c pN0 M0)	**–**	**+**	N/E	N/E	**+**	Figure 2F‐b
27	44	Ovary	Mucinous carcinoma	IIIC (pT3c N0 M0)	**–**	N/A	N/E	N/E	**+**	Figure 2F‐c
28	73	Ovary	High‐grade serous carcinoma	IIIC (pT3c N0 M0)	**–**	N/A	N/E	N/E	**+**	Figure 2F‐d
29	64	Ovary	Poorly differentiated carcinoma	IIIA2 (pT3a pN1 M0)	**–**	**+**	N/E	N/E	N/E	
30	58	Breast	Invasive ductal carcinoma	IIB (pT2 pN1 M0)	**−/+**	**+**	N/E	N/A	N/A	Figure 2G
31	67	Breast	Invasive ductal carcinoma	IA (pT1b pN0 M0)	**+**	**++**	N/E	N/A	N/A	
32	59	Breast	Invasive ductal carcinoma	IIB (pT2 pN1 M0)	**−/+**	**+**	N/E	N/A	N/A	
33	71	Breast	Invasive ductal carcinoma	IIA (pT2 pN0 M0)	**−/+**	**++**	N/E	N/A	N/A	Figure 2H
34	73	Lung	Large cell carcinoma	IIB (pT1c pN1 M0)	**−/+**	**+**	N/E	N/A	N/A	Figure 2I
35	51	Lung	Poorly differentiated NSCLC	IIIA (pT1c pN2 M0)	**–**	**+**	N/E	N/A	N/A	Figure 2J

In endometrial cancers (Table 1), immunohistochemical LVRN expression was negative in myometrial‐invading lesions of primary tumors (Figure 2D(b),(c)), whereas its expression was induced in lymphatic‐invading lesions (Figure 2D(d)–(g)). Similar induction of LVRN expression in lymphovascular lesions was observed in other cases of endometrial cancers (Figure S4A–C, Supporting Information). EpCAM/LVRN double‐positive cells were also observed both in CTCs (Figure S4D, Supporting Information) and ascitic cells from cancerous peritonitis (Figure S4E, Supporting Information).

In various cases of ovarian cancerous peritonitis (Table 1), EpCAM/LVRN double‐positive cancer cells were detected in ascites (Figure 2E). In other 4 cases, LVRN‐positive cancer cells were immunohistochemically observed in peritoneal‐invading lesions (Figure 2F).

In breast (Figure 2G,H) and lung (Figure 2I,J) cancers, a similar increase in LVRN expression was observed in vascular and lymphatic‐invading lesions. In a case of large cell carcinoma of lung cancer, high LVRN expression was observed in the spread through air space (STAS) lesions (Figure 2I(d)). In the other case of adenocarcinoma of lung cancer, tumor cells in the metastatic lymph node were negative for LVRN (Figure 2J(a)), whereas positive tumor cells were observed in efferent lymph vessels (Figure 2J(b)), suggesting that LVRN expression was induced once more in the secondary distant metastatic phase under liquid conditions.

### 2.4 Production of Anti‐LVRN ADC

To examine the potential of anti‐LVRN mAb for clinical application, we conjugated monomethylauristatin E (MMAE, a potent anti‐cancer microtubule‐targeting agent) to control mAb (POG2, mouse anti‐porcine integrin 𝛼6, raised in our laboratory)^[^ ^9^ ^]^ and anti‐LVRN mAb (5‐23) via a valine‐citrulline linker. When MMAE‐conjugated mAbs were internalized into LVRN‐negative HeLa cells using Xfect, both MMAE‐conjugated POG2 (MMAE‐POG2) and 5–23 (MMAE‐5‐23) induced cell death (Figure S5A,B, Supporting Information), showing that MMAE was effectively conjugated with each mAb and adequately released within the cytoplasm of cancer cells. Using these ADCs, we confirmed that MMAE‐5‐23 induced cell death in spheroid‐formed wild‐type A2780 cells, an ovarian cancer cell line, but did not in monolayer‐cultured A2780 cells (Figure S5C–E, Supporting Information).

### 2.5 Anti‐LVRN ADC Induced Cell Death in LVRN‐Positive Ovarian Cancer Cells

**Figure 3:**
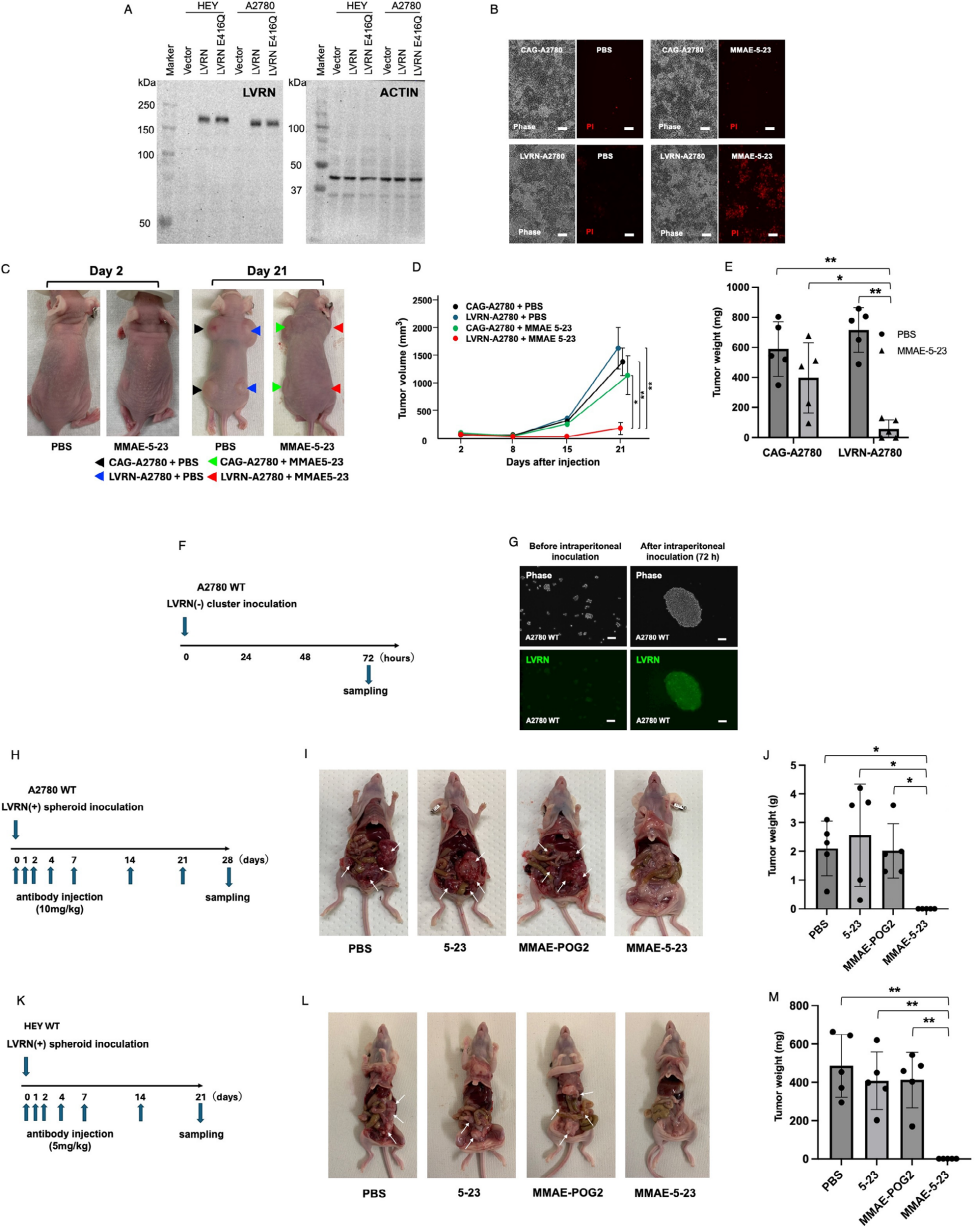
The cytotoxic effects of anti‐LVRN ADC on ovarian cancer cells. A) LVRN and its peptidase‐inactivated mutated form (LVRN E416Q) were transfected into HEY and A2780 cells. B) MMAE‐5‐23 induced cell death in monolayer‐cultured LVRN‐A2780 cells, but not CAG‐A2780 cells. C–E) Intravenous administration of MMAE‐5‐23 significantly inhibited the tumor growth of LVRN‐A2780 cells, but did not inhibit that of CAG‐A2780 cells, both of which were subcutaneously implanted into nude mice. F) Cancer cell clusters of wild‐type A2780 cells (early‐stage spheroid) that were negative for LVRN expression were intraperitoneally inoculated for 72 h. G) Early‐stage spheroids became larger spheroids and acquired LVRN expression. H) Wild‐type A2780 cells were cultured for 72 h to form LVRN‐positive spheroids. Then, they were inoculated into the peritoneal cavity and PBS, 5–23, MMAE‐POG2, or MMAE‐5‐23 were intraperitoneally injected 7 times for 4 weeks. I,J) Intraperitoneal administration of MMAE‐5‐23 significantly inhibited peritoneal dissemination. K–M) Similar anti‐cancer effects of MMAE‐5‐23 were observed in peritoneal dissemination xenograft model mice using wild‐type HEY cells. Bars show 100 µm. Statistical analysis was performed by the One‐way ANOVA with post‐hoc Tukey's multiple comparisons test. ^*^ *p* < 0.05, ^**^ *p* < 0.01. Error bars represent standard deviation.

Then, we generated LVRN‐transfected A2780 (LVRN‐A2780) cells by transfecting them with the pCAG‐GFP‐T2A‐hLVRN‐Puro plasmid, which stably expresses human LVRN and GFP. As a control, we established CAG‐A2780 cells by transfecting them with the pCAG‐GFP‐Puro plasmid, which stably expresses GFP (**Figure** **3**A). MMAE‐5‐23 induced cell death in monolayer‐cultured LVRN‐A2780 cells, but not CAG‐A2780 cells (Figure 3B). When these gene‐transfected A2780 cell lines were subcutaneously implanted into nude mice, intravenous administration of MMAE‐5‐23 significantly inhibited tumor growth of LVRN‐A2780 cells but not that of CAG‐A2780 cells (Figure 3C–E). These findings showed that MMAE‐5‐23 ADC induced LVRN‐specific cell death in the A2780 cells.

### 2.6 Anti‐LVRN ADC Showed Cytotoxic Effects in the Ovarian Cancer Peritoneal Dissemination Model

Then, we examined the cytotoxic effects of MMAE‐5‐23 on wild‐type A2780 cells and another ovarian wild‐type cancer cell line, HEY cells (Figure 1C; Figure S1A,B, Supporting Information), using a peritoneal dissemination mouse xenograft model. First, we confirmed that the cancer cell clusters of A2780 cells (early‐stage spheroid) were initially negative for LVRN, and that they acquired LVRN expression and formed larger spheroids during the 72 h after intraperitoneal inoculation (Figure 3F,G). Next, we cultured wild‐type A2780 cells for 72 h to form spheroids of LVRN‐positive A2780 cells and used them to inoculate the peritoneal cavity. In this model, peritoneal dissemination of wild‐type A2780 cells was established within 28 days, and intraperitoneal administration of MMAE‐5‐23 significantly inhibited their dissemination (Figure 3H–J). Similar anti‐cancer effects of MMAE‐5‐23 were observed in the peritoneal dissemination xenograft model mice using HEY cells (Figure 3K–M).

## 3 Discussion

The floating environment is stressful for epithelial cancer cells, and most CTCs are considered to die from anoikis due to loss of attachment.^[^ ^10^ ^]^ CTCs were also reported to express *POU5F1* and cancer stem cell markers.^[^ ^11^ ^]^ In the liquid phase, some populations become semi‐quiescent for their survival.^[^ ^12^ ^]^ In general, quiescence allows cancer cells to survive in a new environment,^[^ ^13^ ^]^ and quiescent cancer cells are considered resistant to conventional anticancer treatments and contribute to disease recurrence after treatment.^[^ ^14^ ^]^ Therefore, strategies against quiescent cancer stem cells remain important topics,^[^ ^15^ ^]^ and the identification of more effective biomarkers is currently required.^[^ ^16^ ^]^

This study showed that LVRN expression is induced in epithelial cancer cells in the distant metastasizing phase under a floating environment. Recently, LVRN was shown to be a new human embryonic signal that induces IDO1 expression in monocytes/macrophages.^[^ ^5^ ^]^ Importantly, the enzymatic activity of laeverin was not necessary for its immunological effects, suggesting the role of laeverin as a ligand.^[^ ^5^ ^]^ Human chorionic gonadotropin (HCG), secreted by the syncytiotrophoblast,^[^ ^17^ ^]^ and HLA‐G, expressed in the extravillous trophoblast,^[^ ^18^ ^]^ have long been known as human embryonic signals that can regulate the immune environment at the maternal‐fetal interface. However, there are no reports that these two embryonic signals induce IDO1 expression in immune cells. Previously, it has been proposed that HLA‐G expression in malignant tumors is a means of escaping host immune attack,^[^ ^19^ ^]^ and a recent meta‐analysis showed that HLA‐G expression was associated with reduced survival in various solid tumors.^[^ ^20^ ^]^ However, specific roles of HLA‐G and/or HCG in vascular or lymphatic invading cancer cells in the distant metastasizing phase has not been observed. In this regard, the present study is the first to demonstrate the induction of embryonic signals in distant metastasizing epithelial cancer cells.

Spheroid forming culture using cancer cell lines under nonadherent conditions has been an early model of cancer stem cell expansion.^[^ ^21^ ^]^ In this study, LVRN induction by spheroid formation was associated with the expressions of *POU5F1*, suggesting that LVRN is a potential biomarker to identify cancer stem‐like cancer cells in the liquid phase. To support this speculation, the protein with the closest homology to LVRN is a myeloid cell marker, CD13/aminopeptidase N (ANPEP), which also belongs to the M1 peptidase family and has been reported to be a marker for semi‐quiescent human liver cancer stem cells.^[^ ^22^ ^]^

In clinical samples, LVRN expression was observed in vascular and lymphatic‐invasion sites and viable CTCs, confirming that LVRN is specific to cancer cells in the liquid phase and distant metastasizing stages. In contrast, LVRN expression was not detected in the muscular invading lesions prior to lymphovascular invasion, which is compatible with previous report suggesting inhibitory effects of LVRN in cell migration and invasion.^[^ ^23^ ^]^ The additional detection of LVRN expression in STAT lesions of the lung cancer suggests that the non‐adherent state induces LVRN expression in vivo. These findings are consistent with the results that LVRN expression was induced in the spheroid‐formed epithelial cancer cell lines under floating conditions in vitro in culture dishes and in vivo in the mouse peritoneal cavity. Since ovarian cancer cells that had already invaded the sub‐peritoneal regions maintained LVRN expression, it is speculated that LVRN expression continues for some time even after distant metastasis has been established. In contrast to most cancer stem cell markers that are specific to cancer types,^[^ ^24^ ^]^ LVRN induction in the liquid phase was broadly observed in various epithelial cancers, which is consistent with culture experiments. In this regard, LVRN may be a unique cell surface marker that is specific to the liquid phase and applicable to a broader range of epithelial cancers.

ADCs constitute a powerful therapeutic tool for cancer treatment. Their cancer‐killing effects are determined by several factors, such as expression levels of target molecule, the affinity to target molecule, and other molecules that regulate the internalization, linker cleavage, and sensitivity to the cytotoxic agents.^[^ ^25^ ^]^ For example, cathepsins that are highly expressed in various cancers effectively lyses valine‐citrulline linkers to release carcinocidal agents. This study showed that anti‐LVRN mAb (5‐23) was internalized in LVRN‐positive cancer cells and anti‐LVRN ADC, MMAE‐5‐23, can induce their cell death in vitro. Using xenograft models, MMAE‐5‐23 was also shown to effectively inhibit peritoneal dissemination of LVRN‐positive ovarian cancer cells in vivo. In contrast, although 5–23 co‐internalizes LVRN, administration of 5–23 alone without MMAE showed no significant effect on tumor growth in xenograft models implanted with wild‐type cell lines. Since normal immune functions are compromised in xenograft models, further investigations are necessary to properly evaluate the immunological roles of LVRN in tumor growth. Regardless, these findings indicate that LVRN is a unique and promising cell surface target molecule for ADC therapy against liquid‐phase metastasizing cancer cells.

Currently, a number of clinical trials of ADCs targeting cell‐surface molecules such as ERBB2, FOLR1, and NECTIN4 are ongoing. Even when there is a high degree of heterogeneity in the expression of target molecules in solid tumor cells, ADCs can still be effective due to the bystander effect. However, in a floating state, the bystander effect cannot be expected. Meanwhile, several ADC agents that effectively attack cancer stem cells have been proposed for clinical use.^[^ ^26^ ^]^ However, there are few ADC agents that selectively attack epithelial cancer cells, probably including cancer stem‐like cells in the liquid phase. In this regard, anti‐LVRN ADC is a novel candidate for agents that can attack epithelial cancer cells in a floating environment, which have entered the distant metastasizing phase. The detection of LVRN‐positive CTCs and ascitic cancer cells or immunohistochemical observation of LVRN‐positive cancer cells in vascular and lymphatic‐invasion sites may become useful as companion diagnostic biomarkers for patient selection in future ADC trials and for the establishment of clinical criteria to evaluate the indication of anti‐LVRN ADC therapy.

There are several limitations in this study. First, there is insufficient information on the regulatory mechanism of LVRN expression. Since loss of attachment in a suspension environment is stressful for epithelial cancer cells and leads to anoikis, we currently speculate that stress is one of the triggers for LVRN expression in the cancer cells. Elucidation of this mechanism will allow us to develop more efficient anti‐LVRN ADC therapy in the future. Second, since murine LVRN is widely expressed in various organs,^[^ ^4^ ^]^ we have not evaluated the efficacy of anti‐LVRN ADCs against murine cancer cells in mice with normal immune function. These issues should be overcome in the future. Third, this study also lacks information on the molecular biological roles of LVRN in cancer cell functions. Therefore, it should be addressed in the future whether floating cancer cells utilize embryonic signals to regulate their functions, as well as the host's immune system for their own survival. Fourth, the direct effects of anti‐LVRN antibody on cancer cells, including immunological modulation, and its inhibitory effects on hematogenous and lymphatic metastasis also need to be clarified using in vivo mouse models in the future.

In conclusion, this is a pioneering study demonstrating the induction of embryonic signals in distant metastasizing epithelial cancer cells. Currently, clinical application of ADC agents for cancer maintenance chemotherapy is under investigation. From this point of view, anti‐LVRN ADCs are promising agents that can selectively attack cancer cells in the metastatic liquid phase and block further distant metastasis including secondary dissemination, which will provide changes in the clinical approach against distant metastasizing cancer cells. It is also expected that future elucidation of the specific receptor of LVRN will lead to the development of advanced therapeutic approaches based on the ligand‐receptor system. Taking into consideration that LVRN is a trophoblast‐specific molecule, the role of embryonic molecules expressed in cancer cells should be further elucidated from the perspective of both reproductive/perinatal medicine and oncology.

## 4 Experimental Section

### Mice

Balb/c male and Balb/c nu/nu female mice were purchased from SLC Japan. All mice used in this study were housed at the Institute for Experimental Animals at Kanazawa University, in accordance with the institutional guidelines for the care and use of laboratory animals. All protocols were reviewed and approved by the Institutional Animal Care and Use Committee of Kanazawa University (approval Nos. AP‐194088 and AP‐214300). All relevant ethical regulations concerning animal use were complied with.

### Human Tissue Samples

Thirty‐five patients who underwent surgery or multidisciplinary treatment between 2019 and 2023 at Kanazawa University Hospital, Japan, were recruited. Thirty‐five formalin‐fixed paraffin‐embedded blocks (13 cases of cervical, 9 of endometrial, 7 of ovarian, 4 of breast, 2 of lung cancer), 3 ascites samples (1 case of endometrial, 2 of ovarian cancer), and 9 whole‐blood samples (7 cases of cervical, 2 of endometrial cancer) were used. The study protocol was approved by the Medical Ethics Committee of Kanazawa University, and preoperative informed consent was obtained from all patients (approval No. 3267). All ethical regulations relevant to human research participants were followed.

### Cell Lines

Human cancer cell lines were used as follows: melanoma‐ A375 cells (RRID:CVCL_0132) and SKMEL28 cells (RRID:CVCL_0526); cervical cancer‐ SiHa cells (RRID:CVCL_0032), HeLa cells (RRID:CVCL_0030), and C33A cells (RRID:CVCL_1094); ovarian cancer‐ A2780 cells (RRID:CVCL_0134), HEY cells (RRID:CVCL_0297) and SKOV3 cells (RRID:CVCL_0532); endometrial cancer‐ HEC6 cells (RRID:CVCL_2931) and HEC108 cells (RRID:CVCL_2923); prostate cancer‐ DU145 cells (RRID:CVCL_0105), LNCaP cells (RRID:CVCL_0395), and PC3 cells (RRID:CVCL_0035); colon cancer‐ HCT116 cells (RRID:CVCL_0291); lung cancer‐ A549 cells (RRID:CVCL_0023); and breast cancer‐ MCF7 cells (RRID:CVCL_0031) and MDA‐MB‐231 cells (RRID:CVCL_0178). All cell lines were authenticated by STR profiling. Mycoplasma infections were regularly monitored, and all experiments were conducted under mycoplasma‐free conditions.

A375, SKMEL28, SiHa, HeLa, C33A, and DU145 cell lines were cultured in DMEM (Sigma‐Aldrich, USA) containing 10% FBS (Sigma‐Aldrich, USA), and 100 µg mL^−1^ streptomycin/100 IU/mL penicillin (Thermo Fisher Scientific, USA). A2780, SKOV3, LNCaP, PC3, HCT116, and A549 cell lines were cultured in RPMI (Nacalai Tesque, Inc., Japan) containing 10% FBS, and streptomycin/penicillin. HEC6, HEC108, MCF7, and MDA‐MB‐231 cell lines were cultured in DMEM/F12 (Fujifilm Wako Pure Chemical Co., Japan) containing 10% FBS, and streptomycin/penicillin. All cells were cultured at 37 °C with 5% CO_2_.

### Human iPS Cells

iPS cell lines were established using the Stemgent StemRNA‐NM Reprogramming Kit (Reprocell, Japan) following the manufacturer's protocol. Briefly, fibroblasts were cultured in Fibroblast Expansion Medium on iMatrix‐511 (Matrixome, Japan)‐coated dishes on day 1. On days 2 and 4, the RNA Reprogramming cocktail was applied for transfection using RNAiMax Transfection Reagents (Invitrogen, USA). The reprogrammed cells were then cultured in NutriStem Medium (Reprocell, Japan) for 1 to 2 weeks. Primary iPS colonies were picked using a pipette tip, without isolating the surrounding non‐reprogrammed fibroblasts. The reprogrammed cells continued to be cultured until the colonies were fully established. For maintenance and expansion, iPS cells were cultured on iMatrix‐511‐coated dishes and supplemented daily with fresh NutriStem Medium.

For neuronal differentiation, iPS cells were cultured in a differentiation medium composed of Neurobasal Medium (Thermo Fisher Scientific, USA), N2 Supplement (Thermo Fisher Scientific, USA), B27 Supplement (Thermo Fisher Scientific, USA), GlutaMAX Supplement (Thermo Fisher Scientific, USA), 4.5 mg mL^−1^ of glucose (Sigma‐Aldrich, USA), and 100 µg mL^−1^ of streptomycin/100 IU/mL of penicillin. The medium was replaced every other day for 7 days. During differentiation, cells were maintained in a humidified incubator at 37 °C with 5% CO_2_. Neuronal differentiation was confirmed on day 7 via immunocytochemistry using an anti‐TUJ1 antibody (1:500, BioLegend, USA). Non‐fixed cells were harvested for qPCR analysis.

### LVRN Knockdown in Cell Lines

To reduce LVRN expression in cell lines, RNA interference was performed using lentivirus particles containing MISSION small hairpin RNA (LVRN‐shRNA1‐2, Sigma‐Aldrich, USA). Nontarget control lentivirus particles (NC‐shRNA: SHC202V, Sigma‐Aldrich, USA) were used as a negative control. Briefly, A375 cells (1.0 × 10^5^) were seeded in a 12‐well plate. After overnight culture, the cells were infected with lentivirus particles (MOI = 10) in DMEM containing 8 µg mL^−1^ of hexadimethrine bromide (Sigma‐Aldrich, USA). After 20 h, the medium was replaced with fresh culture medium and incubated for another 24 h. The cells were then selected by adding 1 µg mL^−1^ of puromycin (Thermo Fisher Scientific, USA) for 2 weeks.

### LVRN Overexpression in Cell Line

The control plasmid (pCAG‐GFP‐Puro) was created by replacing the flag‐Myc fragment in pCAGIPuro‐FlagmcMycWT (Clone ID: RDB_14 100, RIKEN DNA BANK) with a GFP fragment.^[^ ^27^ ^]^ The hLVRN expression plasmid (pCAG‐GFP‐T2A‐hLVRN‐His‐Puro) was constructed by inserting a cDNA fragment encoding the T2A peptide and full‐length human LVRN tagged with x6 His at the C‐terminus into pCAG‐GFP‐Puro.

A2780 cells (3 × 10^5^) were seeded in a 6‐well plate. After overnight culture, the cells were transfected with either pCAG‐GFP‐Puro or pCAG‐GFP‐T2A‐hLVRN‐His‐Puro plasmid using GenomeOne‐GX (Ishihara Sangyo Kaisha, Ltd., Japan) supplemented with KALA amphipathic peptide (Cosmo Bio Co., Ltd. Japan) according to the manufacturer's protocol. Reagent 1 (3.5 µL), Reagent 2 (21 µL), plasmid (0.2 mg mL^−1^, 21 µL), Reagent 3 (21 µL), KALA (0.02 mg mL^−1^, 21 µL), and Reagent 4 (21 µL) were mixed and incubated on ice for 5 min. The mixture was then added to each well with 2 mL of culture medium, followed by thorough mixing. Enhancer (50 µL) was added and mixed thoroughly again. After 48 h, 1 µg mL^−1^ of puromycin was added to select stable cell lines, which were cultured for one week.

### LVRN Knockout by CRISPR/Cas9 System

Exon 2 of the *LVRN* gene was deleted from the genomic DNA of human cell lines, HCT116 and A549, using the CRISPR/Cas9 system. Specifically, exon 2 was deleted using two gRNAs: gRNA1: CCATGATAATTGGAGTGTGTTGG and gRNA2: AATTAGGTAGAGCAGTCACCTGG, targeting introns 1 and 2, respectively. These gRNAs were separately subcloned into the pX330‐U6‐Chimeric_BB‐CBh‐hSpCas9 plasmid.

4.5 × 10^5^ cells per well were seeded in a 6‐well plate and incubated them overnight. Three plasmid mixes, containing two gRNA and pCAG‐GFP‐Puro, were used to transfect the cells with FuGENE 4K Transfection Reagent (Promega, USA) according to the manufacturer's instructions. After 48 h, the medium was replaced with fresh culture medium. At 3–5 days after transfection, GFP‐positive cells were isolated by FACS and expanded. Gene deletion was confirmed by PCR using the following primers: forward: 5′‐AGTGGAAGATGAAAGCAATGGAC‐3′, and reverse: 5′‐ACTGCCACGTAAGAGCTGTAG‐3′. The PCR conditions were as follows: 94 °C for 60 s, followed by 35 cycles of 30 s at 94 °C, with annealing and extension steps at 60 °C for 30 s and 72 °C for 45 s, respectively. PCR products (WT: 991 bp, KO: 563 bp) were analyzed by running them on a 2% agarose gel. Cells showing knockout bands were selected for single‐cell cloning. Cells were expanded in a confluent state in a 24‐well plate, genotyped by PCR, and stable LVRN knockout cell lines (HCT116‐LVRN‐Cas‐KO and A549‐LVRN‐Cas‐KO) were established.

### Western Blot Analysis

Protein lysates were extracted using RIPA buffer (150 mm NaCl, 50 mm Tris‐HCl (pH 8), 1% NP40, 0.5% deoxycholic acid, and 0.1% SDS) containing a protease inhibitor mixture (Fujifilm Wako Pure Chemical Co., Japan) and phosphatase inhibitor cocktail 1 (Fujifilm Wako Pure Chemical Co., Japan). Each lysate was electrophoresed on 7 or 10% SDS‐PAGE gels and subsequently transferred to PVDF membranes. The membranes were incubated overnight at 4 °C with LVRN rabbit polyclonal antibody (1:1000, raised against human recombinant LVRN in the laboratory)^[^ ^28^ ^]^ or β‐Actin antibody (1:5000, RRID: AB_630 835, Santa Cruz Biotechnology, USA). A secondary horseradish peroxidase‐conjugated antibody was applied for 1 h at room temperature. Visualization of the blots was performed using an enhanced chemiluminescence system and ECL Western Blotting Detection Reagents (GE Healthcare, USA).

### Spheroid Culture

*Shaking Culture Method*: The shaking culture method was applied to generate spheroids from wild‐type cell lines A375, A2780, SiHa, HEC6, HCT116, A549, DU145, SKMEL28, MCF7, MDA‐MB‐231, HEY, SKOV3, HEC108, LNCaP, and PC3. Briefly, 1.0 × 10⁶ cells in 2 mL of culture media were seeded in a well of a Nunclon Sphera‐treated 12‐well plate (Thermo Fisher Scientific, USA). The culture was maintained at an 85‐rpm rotation speed and 5° inclination angle on a bio‐shaker (WEV‐03: AS ONE Co., Japan) at 37 °C with 5% CO_2_ for 72 h. Spheroids were collected with a 1‐mL pipette tip and used for experiments.

*Hanging Drop Method*: The hanging drop method was used to generate spheroids from the WT cell lines A375, A2780, HCT116, and A549. Briefly, 1.0 × 10^3^ cells in 10 µL of culture medium mixed with 10 µg mL^−1^ of FITC‐conjugated anti‐LVRN antibody were seeded on the lid of a culture plate. The cells were cultured for 24 h at 37 °C with 5% CO_2_, and fluorescence was observed.

*Serum‐free Suspension Culture Method*: Spheroids from the A375 cell line were cultured in DMEM/F12 containing 20 ng mL^−1^ of Recombinant Human bFGF (REPROCELL, Japan, Cat. No. RCHEOT002), 20 ng mL^−1^ of Recombinant Human EGF (R&D Systems, USA, Cat. No. 236‐EG), 2% B‐27 Supplement (Thermo Fisher Scientific, USA, Cat. No. 17 504 001), 10 µg mL^−1^ insulin (Sigma‐Aldrich, USA, Cat. No. I5500), and 100 µg mL^−1^ streptomycin/100 IU/mL penicillin. HCT116 and A549 cell lines were cultured in media supplemented with 10 ng mL^−1^ bFGF, 20 ng mL^−1^ EGF, 2% B27 supplement, 4 µg mL^−1^ heparin (STEMCELL Technologies, Canada, Cat. No. 7980), and 100 µg mL^−1^ streptomycin/100 IU/mL penicillin.

For WT cell lines of A375, HCT116, and A549, 1.0 – 2.0 × 10⁵ cells were suspended in 3–4 mL of serum‐free medium and seeded in Nunclon Sphera 6‐well plates. Cultures were maintained for 7 days at 37 °C with 5% CO_2_. For A375 shNC and LVRN KD cell lines (LVRN‐sh1,2), 8.0 × 10⁴ cells were suspended in 2 mL of serum‐free medium and seeded in Nunclon Sphera 12‐well plates. These cells were cultured for 14 days, then split into three wells, and cultured for an additional 7 days.

For WT and LVRN KO of both HCT116 and A549 cell lines, 5.0 × 10⁴ cells were suspended in 1.5 mL of serum‐free medium and seeded in Nunclon Sphera 12‐well plates. Cells were cultured for 7 days, split into three wells, and cultured for an additional 7 days. Medium was refreshed by replacing half the volume every 3 days. For cell splitting, spheroids were disaggregated using 0.05% trypsin (Thermo Fisher Scientific, USA) for 5 min at 37 °C, followed by washing with PBS and pipetting. Spheroids were collected with a 1‐mL pipette tip for experiments.

### Immunohistochemistry

LVRN localization in human specimens was determined using immunohistochemistry with the avidin‐biotin‐peroxidase complex method (VECTASTAIN ABC Kit, Vector Laboratories, Inc., USA) according to the manufacturer's instructions. After deparaffinization, 4‐µm paraffin sections were incubated overnight at 4 °C with anti‐LVRN rabbit polyclonal antibody (1:100). Control staining was performed by replacing the primary antibody with normal rabbit serum. Hematoxylin was used for counterstaining.

### Immunocytochemistry

Spheroids were fixed with 4% PFA for 20 min at room temperature, then incubated with PBS containing 0.5% Triton X‐100 (Sigma‐Aldrich, USA), for 15 min at room temperature. They were subsequently blocked with PBS containing 10% FBS for 1 h at room temperature. For adherent, floating cells, and CTCs, fixation was performed using 4% PFA for 10 min at room temperature, followed by incubation with PBS containing 0.1% Triton X‐100 for 10 min and blocking with PBS containing 10% FBS for 30 min.

All samples were incubated overnight at 4 °C with the primary antibody (10 µg mL^−1^, LVRN mouse monoclonal 5–23). Control staining was performed by replacing the primary antibody with mouse IgG1 isotype control (Proteintech Group, Inc., USA, Cat. No. 65124‐1‐Ig). After washing, the samples were incubated with the secondary antibody (goat anti‐mouse IgG antibody, Alexa Fluor Plus 488, 1:500, Invitrogen, USA, Cat. No. 65124‐1‐Ig) for 1 h at room temperature. Nuclei were stained using Hoechst 33 342 (1:1000, Thermo Fisher Scientific, USA, Cat. No. H1399) for 10 min at room temperature.

### RNA Extraction, Reverse Transcription, and Quantitative Real‐Time PCR (qPCR)

Total RNA was extracted from adherent cells or spheroids using the RNeasy Mini Kit (Qiagen, Germany, Cat. No. 74 104), in accordance with the manufacturer's instructions. The extracted RNA was reverse‐transcribed into cDNA using the PrimeScript RT‐PCR Kit (Takara Bio Inc., Japan, Cat. No. RR014A). qPCR was conducted with specific primers (Table S1, Supporting Information). The PCR cycling conditions were as follows: 95 °C for 30 s, followed by 40 cycles of 5 s at 95 °C, and annealing and extension steps at 60 °C for 30 s, performed using the AriaMx Real‐Time PCR System (Agilent Technologies, USA). *HPRT1* was used as the control gene. Results are presented as the mean ± SD from three independent experiments.

### Analysis of Cell Apoptosis in Spheroid

The apoptosis/live cell ratios of monolayer and spheroid‐cultured A2780 WT cells were evaluated using the Caspase‐Glo 3/7 Assay System (Promega, USA, Cat. No. G8090) and PrestoBlue Cell Viability Reagent. For monolayer cultures, A2780 WT cells (1.0 × 10^3^ cells) were suspended in 100 µL of culture medium and seeded in a 96‐well plate. After 24 h of culture, the medium was replaced with fresh medium containing PBS, POG2 (10 µg mL^−1^), 5–23 (10 µg mL^−1^), MMAE‐POG2 (10 µg mL^−1^), or MMAE‐5‐23 (10 µg mL^−1^). The cells were cultured for an additional 72 h, after which viability and apoptosis were assessed. For the viability assay, 10 µL of PrestoBlue Cell Viability Reagent was added to 100 µL of culture medium, and the cells were incubated for 1 h at 37 °C with 5% CO_2_. Absorbance was subsequently measured using the EnSight multimode plate reader. For the apoptosis assay, 100 µL of Caspase‐Glo 3/7 Reagent was added to 100 µL of culture medium, and the cells were incubated for 30 min at room temperature after gentle shaking at 300 rpm for 30 s. Luminescence was subsequently measured using the EnSight multimode plate reader. For spheroid cultures, A2780 WT cells (1.0 × 10^3^ cells) suspended in 100 µL of culture media containing PBS, POG2 (10 µg mL^−1^), 5–23 (10 µg mL^−1^), MMAE‐POG2 (10 µg mL^−1^), or MMAE‐5‐23 (10 µg mL^−1^) were seeded in Nunclon Sphera 96‐well U‐shaped‐bottom plates and cultured for 72 h. The cells were then subjected to viability and apoptosis assays. The viability of spheroid‐formed cells was also observed using propidium iodide (PI: 50 µg mL^−1^, Sigma‐Aldrich, USA, Cat. No. P4170) staining.

### Intracytoplasmic Internalization of pHrodo‐Labeled Anti‐LVRN mAb

pHrodo dye was conjugated to 5–23 using pHrodo iFL Red Antibody Labeling Kit (Invitrogen, USA, Cat. No. P36020) according to the manufacturer's protocol. A375 WT cells (5.0 × 10^2^ cells) were suspended in 100 µL of culture medium containing pHrodo‐conjugated 5–23 (10 µg mL^−1^) and seeded in a Nunclon Sphera 96‐well U‐shaped‐bottom plate. After 24 h of floating culture at 37 °C with 5% CO_2_, the intracytoplasmic red fluorescence induced by the internalization of pHrodo was observed in the spheroid‐formed A375 cells using a fluorescence microscope.

To further investigate the effect of antibody‐induced LVRN internalization on *POU5F1* expression in spheroid‐formed A375 cells, A375 WT cells (8.0 × 10⁴ cells) suspended in 2 mL of serum‐free medium were seeded in Nunclon Sphera 12‐well plates and cultured for 7 days at 37 °C with 5% CO_2_ to form LVRN‐positive spheroids. Then, half of the medium was replaced with fresh medium, and 5–23 (10 µg mL^−1^) or a control antibody (10 µg mL^−1^) was added. The cells were further cultured for 3 days at 37 °C with 5% CO_2_ and subsequently subjected to quantitative real‐time PCR analysis.

### Detection and Isolation of Circulating Tumor Cells (CTC)

For CTC isolation, 7 mL of blood was collected from cervical or endometrial cancer patients with metastasis. First, PBMCs were isolated from the blood using Ficoll‐Paque Plus (Cytiva, Japan, Cat. No. 17 144 003). PBMCs were fixed with 2% PFA for 10 min at room temperature and blocked with Human BD Fc Block (BD Biosciences, USA, Cat. No. 564 220) for 30 min at room temperature. For CTC detection, both positive and negative selection methods were utilized.

For positive selection, 100 µL of EpCAM (CD326) MicroBeads (Miltenyi Biotec, Germany, Cat. No. 130‐061‐101) was added to 400 µL of PBMCs and incubated for 30 min at 4 °C. Next, 10 µg mL^−1^ LVRN mouse monoclonal antibody 5–23 and CD45 rabbit monoclonal antibody (1:100, Abcam, UK, Cat. No. ab40763) were added as the first antibodies and incubated for 1 h at room temperature. Secondary antibodies (goat anti‐mouse IgG secondary antibody, Alexa Fluor Plus 488, 1:500, Invitrogen, USA, Cat. No. A32723 and goat anti‐rabbit IgG antibody, Alexa Fluor Plus 555, 1:500, Invitrogen, USA, Cat. No. A21429) were then added and incubated for 1 h at room temperature. Hoechst 33 342 (1:1000) was then added to stain the nuclei, followed by 10‐min incubation at room temperature. The cells were separated using the POSSELD mode of autoMACS Pro Separator (Miltenyi Biotec, Germany), and positive fractions were collected. CD45(‐) cells were identified as CTCs.

For negative selection, 20 µL of CD45 MicroBeads (Miltenyi Biotec, Germany, Cat. No. 130‐045‐801) was added to 80 µL of PBMCs and incubated for 15 min at 4 °C. Then, 10 µg mL^−1^ LVRN mouse monoclonal antibody 5–23 and EpCAM rabbit monoclonal antibody (1:100, Abcam, UK, Cat. No. ab71916) were added as the first antibodies and incubated for 1 h at room temperature. Secondary antibodies (goat anti‐mouse IgG antibody, Alexa Fluor Plus 488, 1:500, and goat anti‐rabbit IgG antibody, Alexa Fluor Plus 555, 1:500) were then added and incubated for 1 h at room temperature. BV421‐conjugated anti‐CD45 antibody (1:100, Becton Dickinson, cat no. 563 879) was then added and incubated for 30 min at 4 °C. The cells were separated using the DEPLETE mode of autoMACS Pro Separator, and negative fractions were collected. EpCAM(+) or (−), and CD45(−) cells were identified as CTCs.

### Detection of HPV‐DNA

CTCs prepared from the blood of cervical cancer patients using positive selection were separated into single cells by manual picking with a 20 µm glass capillary unit and UnipicK (Nepagene Inc., Japan). Genomic DNA was then isolated and amplified using the GenomePlex Single Cell Whole Genome Amplification Kit (Sigma‐Aldrich, USA, Cat. No. 89–4771) and purified with the QIAquick PCR Purification Kit (Qiagen, Germany, Cat. No. 28 106), following the manufacturer's instructions. The HPV16 E6/7 gene was detected via PCR using specific primers (forward primer: 5′‐GTGGACCGGTCGATGTATGT‐3′; reverse primer: 5′‐AGATCAGTTGTCTCTGGTTGC‐3′). The PCR cycling conditions consisted of 30 cycles of 30 s at 94 °C, followed by an annealing step at 60 °C for 120 s and extension step at 72 °C for 30 s. PCR products (131 bp) were visualized by electrophoresis on a 4% agarose gel.

### Generation and Purification of Monoclonal Antibodies and Conjugation of MMAE

Hybridomas producing monoclonal antibodies to hLVRN (5‐23/CHL2) ^1^ and POG2 ^11^ were generated in the laboratory as previously reported. Hybridoma cells were cultured with Hybridoma‐SFM (Thermo Fisher Scientific, USA, Cat. No. 12 045 076) at 37 °C with 5% CO_2_.

For large‐scale monoclonal antibody production, 10 male Balb/c mice (11 weeks old) were used. First, 500 µL of pristane (FUJIFILM Wako Pure Chemical Co., Japan, Cat. No. 163–27482) was injected intraperitoneally per mouse. One week after injection, 1.0 × 10^7^ hybridoma cells suspended in PBS were injected intraperitoneally per mouse. At 10 to 14 days after cell implantation, ascites was collected from mice if present through 18 or 20‐gauge needles and collected in 50‐mL conical tubes. After centrifugation at 1000 rpm for 5 min at 4 °C, the supernatant was collected, mixed with an equivalent volume of saturated ammonium sulfate solution, and stored at 4 °C. These ascites were purified with Protein A (Hokudo Co., Ltd., Japan).

Monoclonal antibodies were conjugated to monomethylauristatin E (MMAE) through a valine‐citrulline linker.^[^ ^29^ ^]^ Briefly, monoclonal antibodies (20 mg) in PBS containing 10 mm Tris buffer (pH 8.5) were reduced with 2.5 equiv. tris (2‐carboxyethyl) phosphine hydrochloride (TCEP‐HCl; FUJIFILM Wako Pure Chemical Co., Japan, Cat. No. 205–19863) at 26 °C for 1 h. The reduced antibodies were incubated with 10 equiv. maleimidocaproyl‐valine‐citrulline‐p‐aminobenzoyloxycarbonyl MMAE (MC‐Val‐Cit‐PAB‐MMAE; Med Chem Express, USA, Cat. No. HY‐15575) in 20% v/v acetonitrile at 26 °C for 1 h. Unconjugated payload was removed by ultrafiltration (Cut MW: 100000; Merck Millipore, USA, Cat. No. PBHK02510) with extensive buffer substitution to PBS. Protein concentration was determined by the bicinchoninic acid assay (Nacalai Tesque, Inc., Japan, Cat. No. 06385‐00) using bovine immunoglobulin G as a standard.

To verify the success of MMAE conjugation, the MMAE‐conjugated antibody was used to transfect the HeLa WT cells using Xfect Protein Transfection Reagent (Clontech Laboratories, Inc., USA, Cat. No. Z1323N). Briefly, cells were seeded in 24‐well plates at 5 × 10^4^ cells per well the day before transfection. Then, 10 µg of MMAE‐conjugated antibody was used for transfection according to the manufacturer's instructions. β‐Galactosidase was used as a positive control. At 72 h after transfection, cell viability was monitored by PI staining.

To determine whether the MMAE‐anti‐hLVRN antibody is cytotoxic to A2780 WT spheroids expressing LVRN, 1.0 × 10^5^ A2780 WT cells were suspended in 2 mL of culture medium containing PBS, 10 µg mL^−1^ of MMAE‐POG2, or 10 µg mL^−1^ of MMAE‐5‐23 and seeded in Nunclon Sphera 12‐well plates. Cell viability was observed by PI staining 72 h after seeding.

### Xenograft Models

To generate a xenograft model, two cell lines were used: CAG‐A2780 and LVRN‐A2780. The CAG‐A2780 cell was established by transfection with pCAG‐GFP‐Puro plasmid stably expressing GFP. The LVRN‐A2780 cell was established by transfection with pCAG‐GFP‐T2A‐hLVRN‐Puro plasmid stably expressing hLVRN and GFP. The cells (5.0 × 10^6^) suspended in 100 µL of PBS were inoculated subcutaneously into 6 to 8 weeks old Balb/c nu/nu female mice. Then, 100 µL of PBS or MMAE‐conjugated 5–23 suspended in PBS (10 mg/kg bw) were injected through the tail vein 2 and 8 days after cancer cell inoculation. Tumor size and body weight were measured once a week. Tumor volume was calculated as 0.5 x width (mm) x length (mm)^2^.

To verify whether A2780 WT cells express LVRN after intraperitoneal inoculation, we intraperitoneally inoculated nude mice with them. Briefly, 1.0 × 10^6^ A2780 cells were suspended in 3 mL of culture medium and seeded in Nunclon Sphera 6‐well plates. After 12 h of incubation, cancer cell clusters (early‐stage spheroids) were collected with a 1‐mL pipette tip and washed with PBS. Then, clusters (early‐stage spheroids) of 1.0 × 10^6^ cells were suspended in 300 µL of PBS and used to inoculate intraperitoneally 8‐week‐old Balb/c nu/nu female mice. After making a small incision in the abdominal skin and separating it from the peritoneum, a small incision was made in the peritoneum, and cell clusters were injected into the peritoneum with a 1‐mL pipette tip. The peritoneal wound was closed with sutures, and skin was closed with wound clips. After 72 h, intraperitoneal cancer cell spheroids were collected by washing with 10 mL of PBS and used for immunocytochemistry.

To generate another model, spheroids were used to intraperitoneally inoculate nude mice. Briefly, A2780 cells (3.0 × 10^3^ cells) or HEY cells (3.0 × 10^3^ cells) were suspended in 100 µL of culture medium and seeded in Nunclon Sphera 96‐well U‐shaped‐bottom plates. After 72 h of incubation, spheroids were collected with a 1‐mL pipette tip and washed with PBS. Spheroids from 30 wells were then suspended in 300 µL of PBS with or without 5–23, MMAE‐POG2, or MMAE‐5‐23 (10 mg/kg bw for A2780 cells and 5 mg/kg bw for HEY cells) and used to intraperitoneally inoculate 8‐week‐old Balb/c nu/nu female mice. After making a small incision in the abdominal skin and separating it from the peritoneum, a small incision was made in the peritoneum and spheroids were injected into the peritoneum with a 1‐mL pipette tip. The peritoneal wound was closed with sutures, and skin was closed with wound clips. At 1, 3, 5, 7, 14, and 21 days after inoculation, mice were injected peritoneally with 50 µL of PBS with or without 5–23, MMAE‐POG2, or MMAE‐5‐23 (10 mg/kg bw for A2780 cells and 5 mg/kg bw for HEY cells). Body weight was monitored once a week. At 21 or 28 days after inoculation, tumors were harvested to measure their weight (g).

### Statistical Analysis

All statistical analyses were performed with GraphPad Prism 10 software. The normality of values in each group was confirmed using the Shapiro‐Wilk normality test. One‐way analysis of variance (ANOVA) with post hoc Tukey's multiple comparison test was performed to compare the means between each experimental group. Student's t‐test was used to compare means between the two experimental groups. The sample size is indicated in the legend, and no sample was excluded from analysis. P value < 0.05 was considered significant in this study.

## Conflict of Interest

H.F. is listed in a patent application related to this work.

## Author Contributions

H.K., Y.S., and K.K. contributed equally to this work. K.K., A.H., T.D., and H.F. conceived the study and study design; H.K., Y.S., K.K., T.I., T.K., T.S., K.K., A.H., and T.D. performed the experiments and data analysis; H.K., Y.S., T.I., T.K., S.Y., N.I., I.M., and R.Y. prepared clinical samples; H.K., M.O., T.D., N.M., and H.F. wrote the paper; M.O., K.A., N.M., A.H., T.D., and H.F. discussed the paper; T.F., M.O., T.D., and H.F. contributed to funding acquisition; H.F. was the originator of the concept of this report. All authors approved this paper.

## Supporting information



Supporting Information

## Data Availability

The data that support the findings of this study are available from the corresponding author upon reasonable request.

## References

[1] H. Fujiwara , T. Higuchi , S. Yamada , T. Hirano , Y. Sato , Y. Nishioka , S. Yoshioka , K. Tatsumi , M. Ueda , M. Maeda , S. Fujii , Biochem. Biophys. Res. Commun. 2004, 313, 962. 14706636 10.1016/j.bbrc.2003.12.024

[2] M. Maruyama , A. Hattori , Y. Goto , M. Ueda , M. Maeda , H. Fujiwara , M. Tsujimoto , J. Biol. Chem. 2007, 282, 20088. 17525158 10.1074/jbc.M702650200

[3] A. C. Staff , H. E. Fjeldstad , I. K. Fosheim , K. Moe , G. Turowski , G. M. Johnsen , P. Alnaes‐Katjavivi , M. Sugulle , Am. J. Obstet. Gynecol. 2022, 226, S895. 32971013 10.1016/j.ajog.2020.09.026

[4] T. Tobita , D. Kiyozumi , M. Muto , T. Noda , M. Ikawa , J. Reprod. Dev. 2019, 65, 239. 30745494 10.1262/jrd.2018-157 PMC6584185

[5] T. Suzuki , T. Iizuka , K. Kagami , T. Matsumoto , R. Yamazaki , T. Daikoku , A. Horie , M. Ono , A. Hattori , H. Fujiwara , iScience 2023, 26, 107692. 37705960 10.1016/j.isci.2023.107692 PMC10495628

[6] A. Horie , H. Fujiwara , Y. Sato , K. Suginami , H. Matsumoto , M. Maruyama , I. Konishi , A. Hattori , Hum. Reprod. 2012, 27, 1267. 22402206 10.1093/humrep/des068

[7] T. Kanda , K. Kagami , T. Iizuka , H. Kasama , T. Matsumoto , Y. Sakai , T. Suzuki , M. Yamamoto , A. Matsuoka , R. Yamazaki , A. Hattori , A. Horie , T. Daikoku , M. Ono , H. Fujiwara , Am. J. Reprod. Immunol. 2023, 90, 13752. 10.1111/aji.13752 37491922

[8] S. L. Ham , R. Joshi , P. S. Thakuri , H. Tavana , Exp. Biol. Med. 2016, 241, 939. 10.1177/1535370216643772 PMC4950350 27072562

[9] H. Fujiwara , M. Ueda , K. Takakura , T. Mori , M. Maeda , Biol. Reprod. 1995, 53, 407. 7492694 10.1095/biolreprod53.2.407

[10] G. Follain , D. Herrmann , S. Harlepp , V. Hyenne , N. Osmani , S. C. Warren , P. Timpson , J. G. Goetz , Nat. Rev. Cancer 2020, 20, 107. 31780785 10.1038/s41568-019-0221-x

[11] R. Zhang , J. Xia , Y. Wang , M. Cao , D. Jin , W. Xue , Y. Huang , H. Chen , Onco. Targets Ther. 2020, 13, 10739. 33122913 10.2147/OTT.S259240 PMC7588836

[12] C. Wenzel , B. Riefke , S. Grundemann , A. Krebs , S. Christian , F. Prinz , M. Osterland , S. Golfier , S. Rase , N. Ansari , M. Esner , M. Bickle , F. Pampaloni , C. Mattheyer , E. H. Stelzer , K. Parczyk , S. Prechtl , P. Steigemann , Exp. Cell Res. 2014, 323, 131. 24480576 10.1016/j.yexcr.2014.01.017

[13] A. Recasens , L. Munoz , Trends Pharmacol. Sci. 2019, 40, 128. 30612715 10.1016/j.tips.2018.12.004

[14] E. Batlle , H. Clevers , Nat. Med. 2017, 23, 1124. 28985214 10.1038/nm.4409

[15] Y. Ohta , M. Fujii , S. Takahashi , A. Takano , K. Nanki , M. Matano , H. Hanyu , M. Saito , M. Shimokawa , S. Nishikori , Y. Hatano , R. Ishii , K. Sawada , A. Machinaga , W. Ikeda , T. Imamura , T. Sato , Nature 2022, 608, 784. 35798028 10.1038/s41586-022-05043-y

[16] X. Chu , W. Tian , J. Ning , G. Xiao , Y. Zhou , Z. Wang , Z. Zhai , G. Tanzhu , J. Yang , R. Zhou , Signal Transduct. Target Ther. 2024, 9, 170. 38965243 10.1038/s41392-024-01851-y PMC11224386

[17] A. Schumacher , A. C. Zenclussen , Front. Immunol. 2019, 10, 2896. 31921157 10.3389/fimmu.2019.02896 PMC6914810

[18] B. Zhuang , J. Shang , Y. Yao , Front. Immunol. 2021, 12, 744324. 34777357 10.3389/fimmu.2021.744324 PMC8586502

[19] N. Rouas‐Freiss , P. Moreau , S. Ferrone , E. D. Carosella , Cancer Res. 2005, 65, 10139. 16287995 10.1158/0008-5472.CAN-05-0097

[20] J. Bartolome , C. Molto , J. D. Benitez‐Fuentes , G. Fernandez‐Hinojal , A. Manzano , P. Perez‐Segura , A. Mittal , F. Tamimi , E. Amir , A. Ocana , Front. Immunol. 2023, 14, 1165813. 37275862 10.3389/fimmu.2023.1165813 PMC10232772

[21] L. B. Weiswald , D. Bellet , V. Dangles‐Marie , Neoplasia 2015, 17, 1. 25622895 10.1016/j.neo.2014.12.004 PMC4309685

[22] N. Haraguchi , H. Ishii , K. Mimori , F. Tanaka , M. Ohkuma , H. M. Kim , H. Akita , D. Takiuchi , H. Hatano , H. Nagano , G. F. Barnard , Y. Doki , M. Mori , J. Clin. Invest. 2010, 120, 3326. 20697159 10.1172/JCI42550 PMC2929722

[23] X. Feng , Z. Wei , S. Zhang , J. Zhou , J. Wu , B. Luan , Y. Du , H. Zhao , Acta Biochim. Biophys .Sin 2021, 53, 249. 33355358 10.1093/abbs/gmaa167

[24] M. A. Sarabia‐Sanchez , J. M. Tinajero‐Rodriguez , E. Ortiz‐Sanchez , E. Alvarado‐Ortiz , Life Sci. 2024, 355, 123015. 39182567 10.1016/j.lfs.2024.123015

[25] C. Bosi , A. Bartha , B. Galbardi , G. Notini , M. M. Naldini , L. Licata , G. Viale , M. Mariani , B. Pistilli , H. R. Ali , F. Andre , M. Piras , M. Callari , M. Barreca , A. Locatelli , L. Vigano , C. Criscitiello , L. Pusztai , G. Curigliano , B. Gyorffy , M. Dugo , G. Bianchini , Eur. J. Cancer 2023, 195, 113379. 37913680 10.1016/j.ejca.2023.113379

[26] Y. Hu , Y. Zhu , D. Qi , C. Tang , W. Zhang , Biomark Res. 2024, 12, 82. 39135109 10.1186/s40364-024-00633-6 PMC11321197

[27] H. Iseki , Y. Nakachi , T. Hishida , Y. Yamashita‐Sugahara , M. Hirasaki , A. Ueda , Y. Tanimoto , S. Iijima , F. Sugiyama , K. Yagami , S. Takahashi , A. Okuda , Y. Okazaki , Stem Cells 2016, 34, 322. 26523946 10.1002/stem.2243

[28] M. Maruyama , N. Arisaka , Y. Goto , Y. Ohsawa , H. Inoue , H. Fujiwara , A. Hattori , M. Tsujimoto , J. Biol. Chem. 2009, 284, 34692. 19819873 10.1074/jbc.M109.066712 PMC2787332

[29] S. O. Doronina , B. E. Toki , M. Y. Torgov , B. A. Mendelsohn , C. G. Cerveny , D. F. Chace , R. L. DeBlanc , R. P. Gearing , T. D. Bovee , C. B. Siegall , J. A. Francisco , A. F. Wahl , D. L. Meyer , P. D. Senter , Nat. Biotechnol. 2003, 21, 778. 12778055 10.1038/nbt832

