# Supplementary material for: Laeverin is Cell‐Surface Target for Liquid‐Phase Metastasizing Cancer Cells
Source: Adv Sci (Weinh). 2025 Sep 29;12(47):e11349. doi: 10.1002/advs.202511349 (PMC12713063; doi:10.1002/advs.202511349)
Supplement: Supplementary file 1 — Supporting Information [file ADVS-12-e11349-s001.docx]

**Supporting information**

**Advanced Science**

**Laeverin is Cell-surface Target for Liquid-phase Metastasizing Cancer Cells**

Haruki Kasama^1†^, Yuya Sakai^1†^, Kyosuke Kagami^1†^, Takashi Iizuka^1^, Tatsuhito Kanda^1^, Takuma Suzuki^1^, Kayo Kayahashi^1^, Masanori Ono^2^, Tomoko Fujiwara^3^, Shintaro Yagi^4^, Noriyuki Inaki^5^, Isao Matsumoto^6^, Rena Yamazaki^1^, Kaoru Abiko^1^, Noriomi Matsumura^7^, Akira Hattori^8^***, Takiko Daikoku^9^**, and Hiroshi Fujiwara^1,10,11^*

**Supporting information list**

**•** Supporting Table S1

**•** Legends for Supporting Figures S1-S5

**Supporting Table S1. List of primers used in this study**

Primers for quantitative real-time PCR

| Gene name | Forward (5'-3') | Reverse (5'-3') |
| --- | --- | --- |
| *LVRN* | TGAGCAAGATGATCTATGGAGGC | GCTCCTGTTTCATGACGCCAGT |
| *POU5F1* | CTTGCTGCAGAAGTGGGTGGAGGAA | CTGCAGTGTGGGTTTCGGGCA |
| *TUBB3* | TCAGCGTCTACTACAACGAGGC | GCCTGAAGAGATGTCCAAAGGC |
| *HPRT1* | GCCCTGGCGTCGTGATTAGT | CGAGCAAGACGTTCAGTCCTGTC |

Primers for reverse transcription PCR

| Gene name | Forward (5'-3') | Reverse (5'-3') |
| --- | --- | --- |
| *LVRN* | CATTTGCCAGGTATGTTTTCCC | TGCCCCTTTCTGTTCTGTTGAC |
| *POU5F1* | GCTCGAGAAGGATGTGGTCC | CGTTGTGCATAGTCGCTGCT |
| *GAPDH* | AGGGCTGCTTTTAACTCTGGT | CCCCACTTGATTTTGGAGGGA |

**
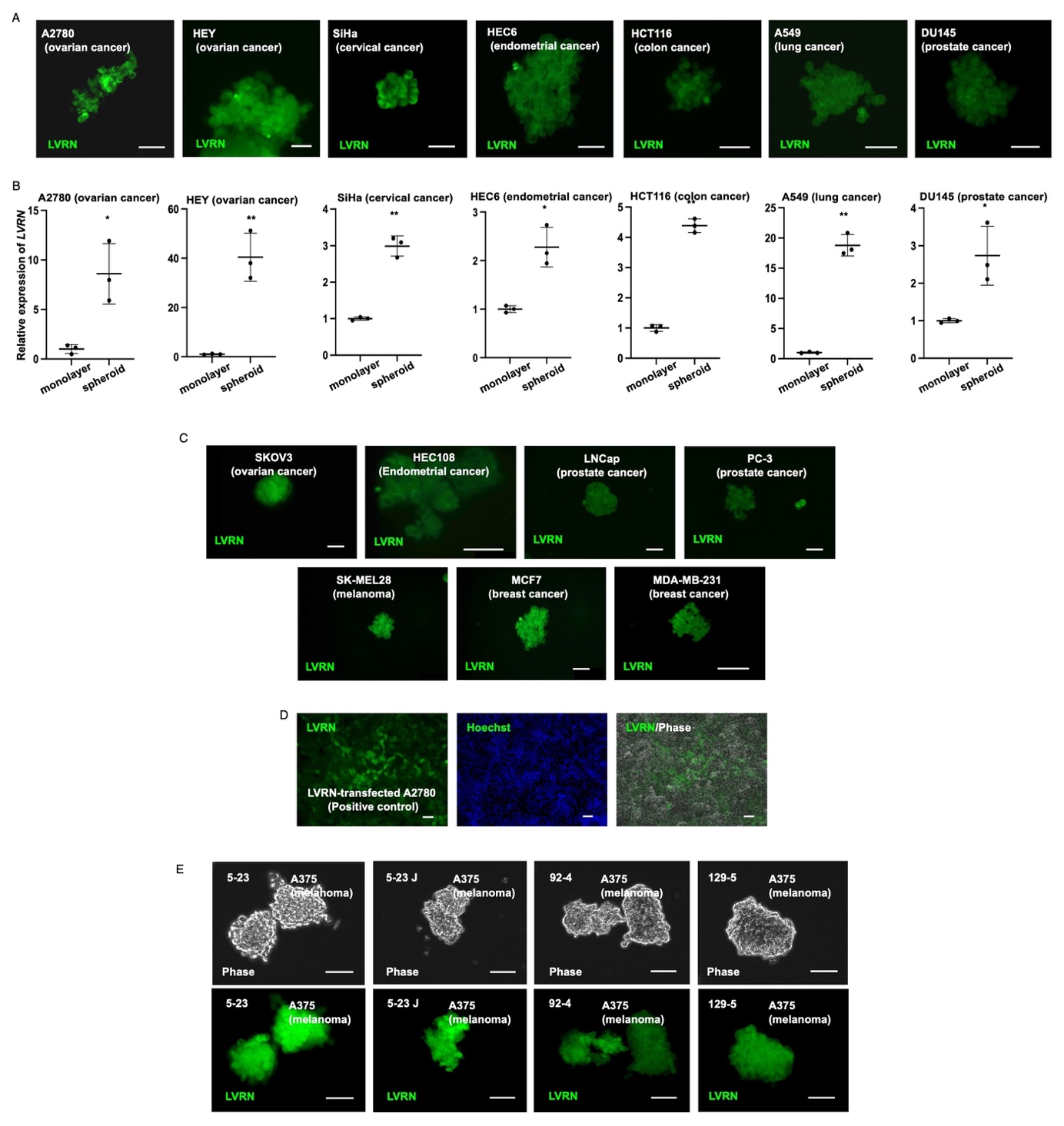
**

**Supporting Figure S1. LVRN expression in the spheroid-forming cancer cell lines**

**A**-**C**. Spheroids of various human epithelial cancer cell lines were prepared using the shaking culture method for 72 h. By immunocytochemical staining using 5-23, the induction of cell surface expression of LVRN was detected (**A** and **C**). **B**. mRNA expression of LVRN was induced by spheroid culture in various cancer cell lines (**A,** corresponding to Fig. **1C**). **D**. Monolayer-cultured LVRN-transfected A2780 cells as positive control. LVRN staining (left panel), Hoechst staining (middle panel), and merged image (right panel). **E**. Similarly positive expression of LVRN on spheroid-forming wild-type A375 cells was detected by each of the different anti-LVRN mAb clones, 5-23, 5-23J, 92-4, 129-5. Bars show 100 μm (C and D) and 50 μm (A and E).


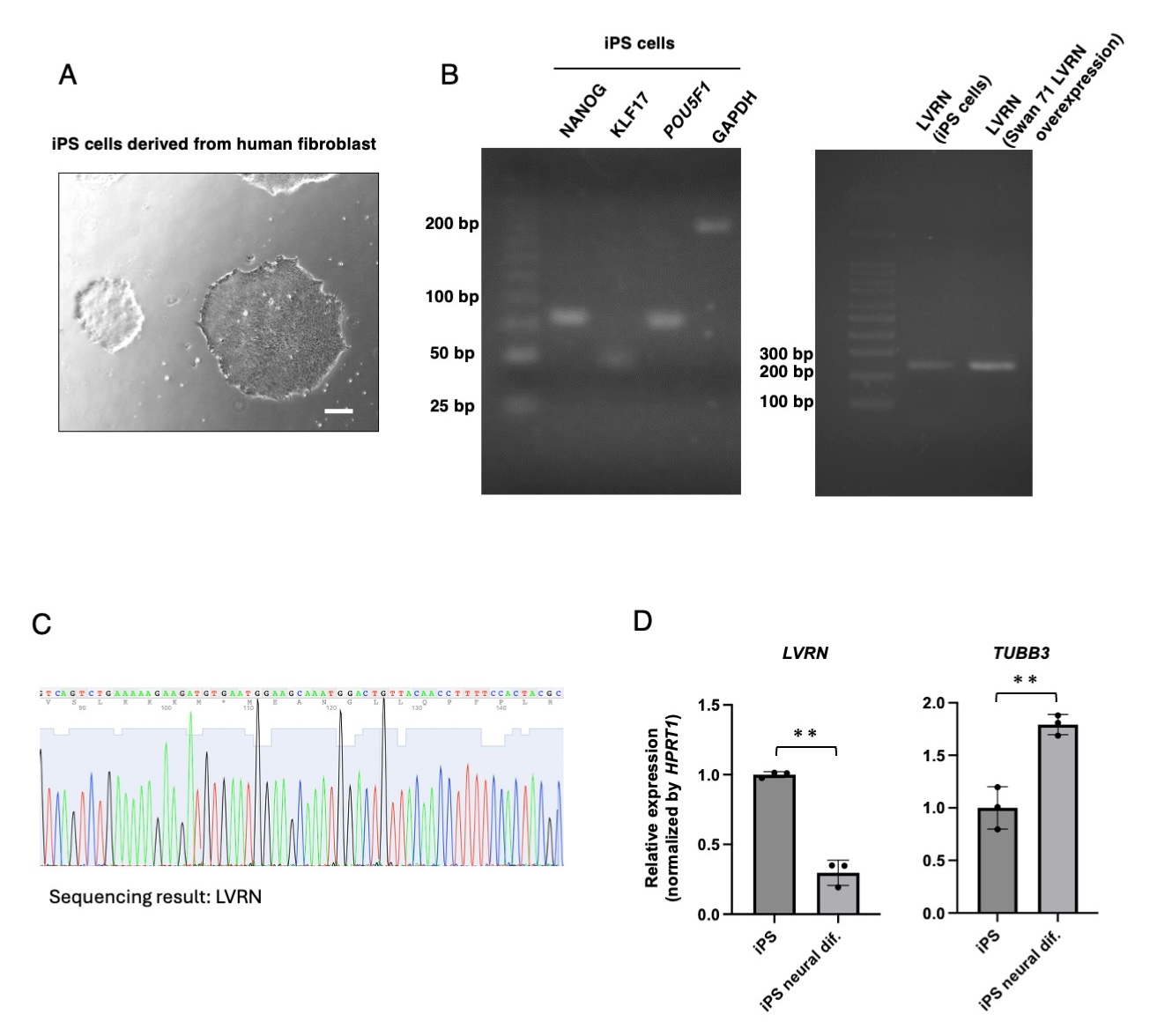


**Supporting Figure S2. LVRN expression in human iPS cells**

**A**. Bright-field image of iPS cells derived from human fibroblasts. Bars show 200 μm. **B**. The expression of iPS-inducing genes and LVRN were detected in iPS cells by RT-PCR. GAPDH: a loading control. LVRN-transfected Swan 71: a positive control. **C**. The sequence of the RT-PCR product of LVRN was revealed to be identical to LVRN. **D.** In iPS cells, LVRN mRNA expression was reduced during neural differentiation culture, where beta Tubulin 3 (TUBB3) expression increased. Statistical analysis was performed by Student’s t-test. ***p*<0.01. Error bars represent standard deviation.

**
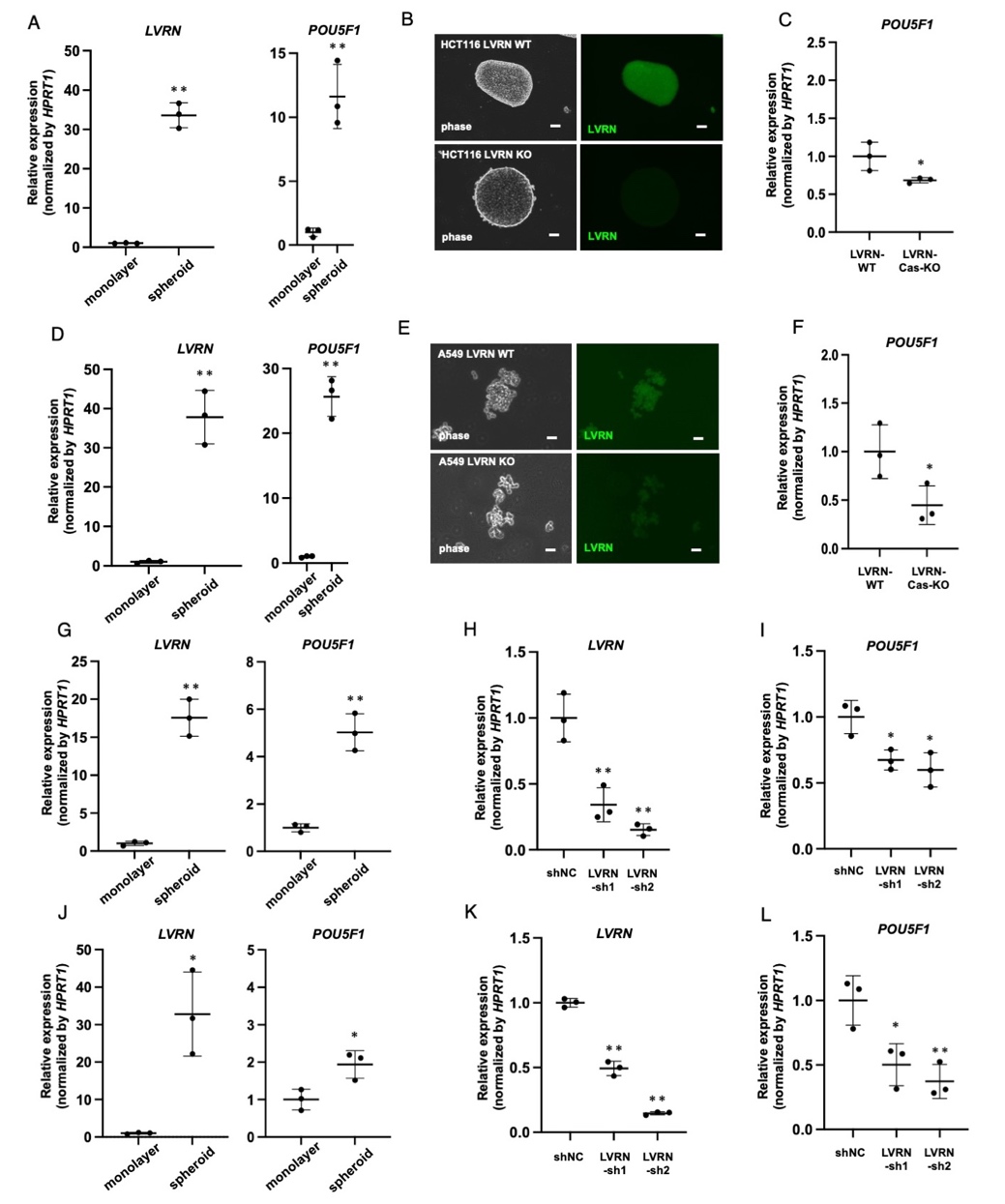
**

**Supporting Figure S3. *POU5F1* expression associated with LVRN in the spheroid-forming HCT116, A549, A2780, and HEY cells**

**A-C**. Spheroid-formed wild-type (WT) and LVRN-knocked out (LVRN-Cas-KO) colorectal cancer-derived HCT116 cells. **A**. Spheroid formation increased *LVRN* and *POU5F1*expressions. **B**. LVRN expression was not induced in HCT116-LVRN-Cas-KO cells. **C**. In the spheroid-formed HCT116-LVRN-Cas-KO cells, *POU5F1* expression significantly decreased. **D-F**. Spheroid-formed wild-type and LVRN-knocked out (LVRN-Cas-KO) lung cancer-derived A549 cells. **D.** Spheroid formation increased *LVRN* and *POU5F1*expressions. **E**. LVRN expression was not induced in the spheroid of A549-LVRN-Cas-KO cells. **F**. In the spheroid-formed A549-LVRN-Cas-KO cells, *POU5F1* expression significantly decreased. **G-I**. Spheroid-formed wild-type and LVRN-knocked down ovarian cancer-derived A2780 cells. **G**. Spheroid formation increased both *LVRN* and *POU5F1* expressions. **H** and **I**. Reductions of *LVRN* expression by shRNAs induced decreases in *POU5F1* expression. **J-L**. Spheroid-formed wild-type and LVRN-knocked down ovarian cancer-derived HEY cells. **J**. Spheroid formation increased both *LVRN* and *POU5F1* expressions. **K** and **L**. Reductions of *LVRN* expression by shRNAs decreased *POU5F1* expression. Statistical analysis was performed by the t-test. Error bars represent standard deviation. **p*<0.05, ** *p* <0.01. Bars show 100 μm.

**
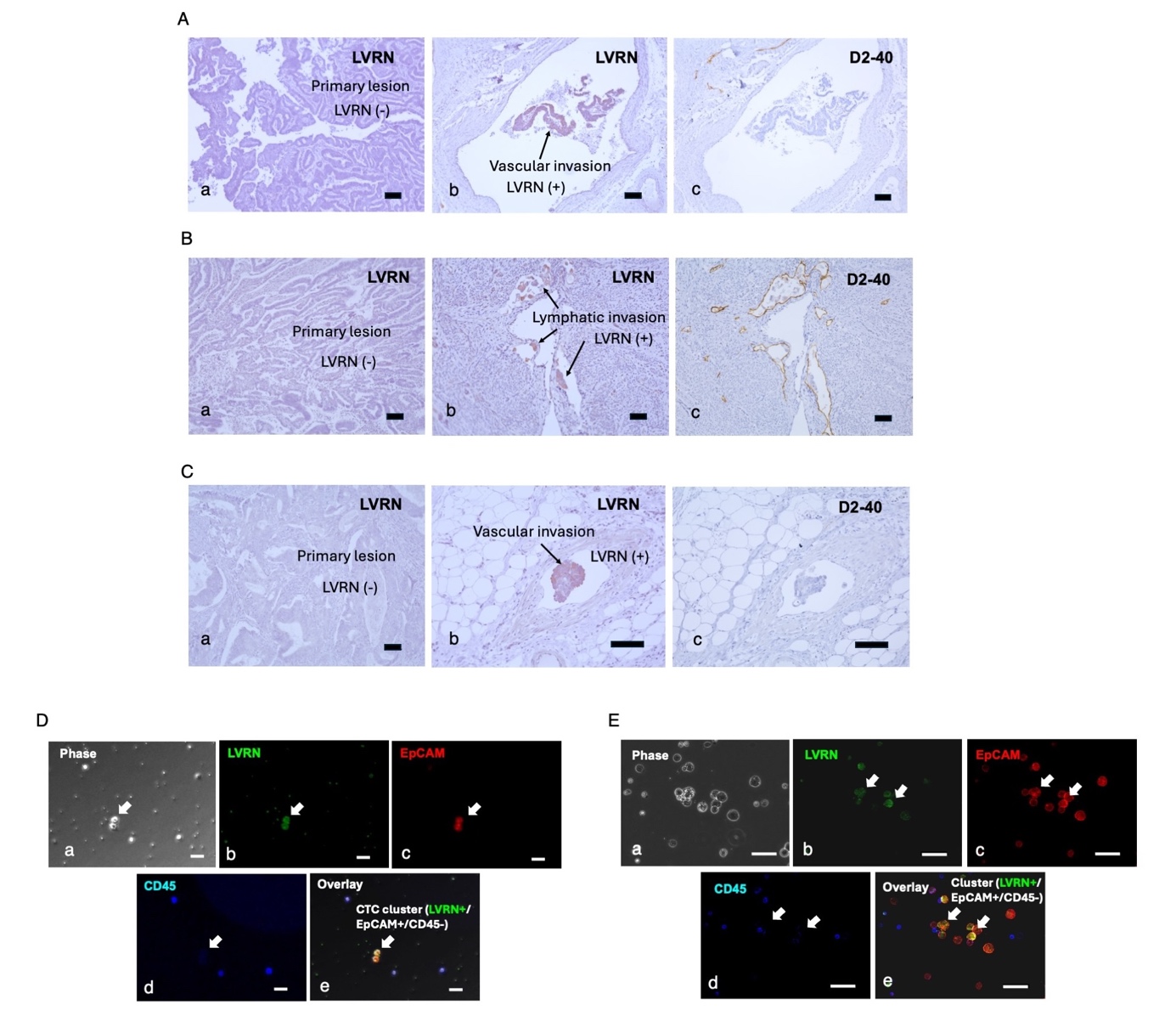
**

**Supporting Figure S4. LVRN expression in other cases of endometrial cancers**

**A-C**. Immunohistochemical expression of LVRN in 3 cases of endometrial cancer. **A**. Endometrioid carcinoma G2, stage ⅠB (T1bN0N0). **B**. Endometrioid carcinoma G1, stage IIIC2 (pT1bN2M0). **A** & **B**. Primary lesion was negative for LVRN (**a**), but lymphovascular-invading lesions in the myometrium were positive for LVRN (**b**). **C**. Endometrioid carcinoma G1, stage IIIC2 (pT1bN2M0). Primary lesion was negative for LVRN (**a**), whereas positive LVRN expression was observed in cancer cells in the vessel adjacent to metastatic lymph node (**b**). Bars show 100 μm (**a**), 50 μm (**b**). In other endometrial cancer patients, EpCAM/LVRN double-positive cells were observed in CTCs (**D**) and ascitic cells (**E**). Bars show 20 μm.


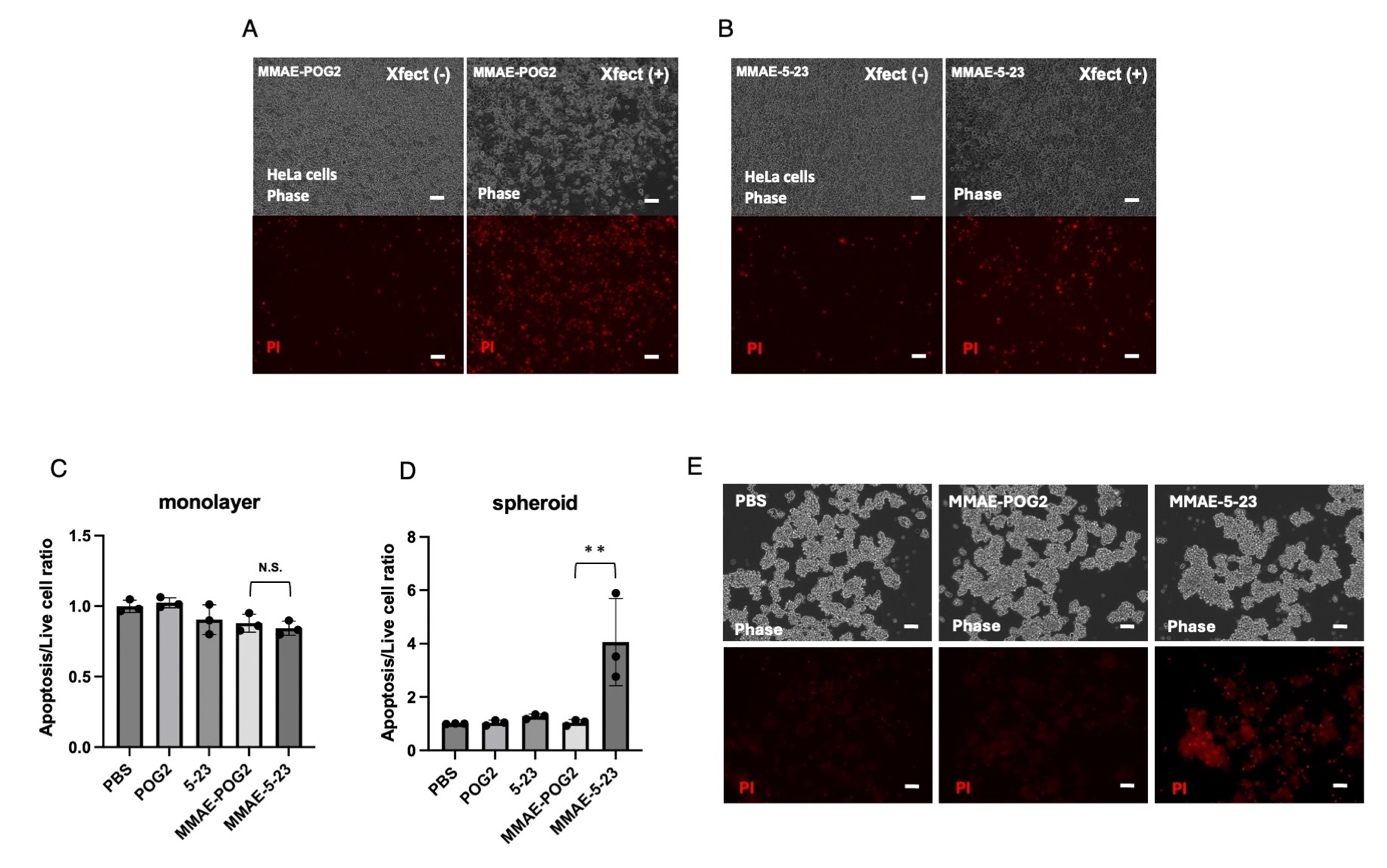


**Supporting Figure S5. Production of anti-LVRN ADC**

**A** & **B**. MMAE was conjugated to control mAb, POG2 (MMAE-POG2) and 5-23 (MMAE-5-23) via valine-citrulline linker. When these MMAE-conjugated mAbs were transfected to LVRN-negative HeLa cells using Xfect^TM^, both MMAE-POG2 (**A**) and MMAE-5-23 (**B**) induced cell death. **C**-**E**. MMAE-5-23 induced cell death in spheroid-formed wild-type A2780 cells, an ovarian cancer cell line (**D** & **E**), but not monolayer-cultured A2780 cells (**C**). Bars show 100 μm. Statistical analysis was performed by the One-way ANOVA with post-hoc Tukey’s multiple comparisons test. **p*<0.05, ***p*<0.01. Error bars represent standard deviation.
